# Endocytosis of nutrient transporters in fungi: The ART of connecting signaling and trafficking

**DOI:** 10.1016/j.csbj.2021.03.013

**Published:** 2021-03-19

**Authors:** Cláudia Barata-Antunes, Rosana Alves, Gabriel Talaia, Margarida Casal, Hernâni Gerós, Robert Mans, Sandra Paiva

**Affiliations:** a Centre of Molecular and Environmental Biology, Department of Biology, University of Minho, Braga, Portugal; b Department of Cell Biology, Yale University School of Medicine, New Haven, CT, United States; c Centre for the Research and Technology of Agro-Environmental and Biological Sciences, University of Trás-os-Montes and Alto Douro, Vila Real, Portugal; d Centre of Biological Engineering (CEB), Department of Biological Engineering, University of Minho, Braga, Portugal; e Department of Biotechnology, Delft University of Technology, Delft, the Netherlands

**Keywords:** AAs, amino acids, ACT, amino Acid/Choline Transporter, AP, adaptor protein, APC, amino acid-polyamine-organocation, Arg, arginine, Arts, arrestin‐related trafficking adaptors, Asp, aspartic acid, C, carbon, C-terminus, carboxyl-terminus, Cu, copper, DUBs, deubiquitinating enzymes, EMCs, eisosome membrane compartments, ER, endoplasmic reticulum, ESCRT, endosomal sorting complex required for transport, fAATs, fungal AA transporters, Fe, iron, GAAC, general amino acid control, Glu, glutamic acid, H^+^, proton, IF, inward-facing, LAT, L-type Amino acid Transporter, LID, loop Interaction Domain, Lys, lysine, MCCs, membrane compartments containing the arginine permease Can1, MCPs, membrane compartments of Pma1, Met, methionine, MFS, major facilitator superfamily, Mn, manganese, MVB, multi vesicular bodies, N, nitrogen, NAT, nucleobase Ascorbate Transporter, NCS1, nucleobase/Cation Symporter 1, NCS2, nucleobase cation symporter family 2, NH_4_^+^, ammonium, N-terminus, amino-terminus, OF, outward-facing, PEST, proline (P), glutamic acid (E), serine (S), and threonine (T), PM, plasma membrane, PVE, prevacuolar endosome, TGN, trans-Golgi network, TMSs, transmembrane segments, TORC1, target of rapamycin complex 1, Trp, tryptophan, TRY, titer, rate and yield, Tyr, tyrosine, Ub, ubiquitin, VPS, vacuolar protein sorting, W/V, weight per volume, YAT, yeast Amino acid Transporter, Zn, Zinc, Fungi, Nutrient transporters, Endocytosis, Arrestins, Ubiquitylation, Endocytic signals, Metabolism, Signaling pathways, Conformational changes, MCCs/eisosomes, Structure-function, Biotechnology, Cell factories, *Saccharomyces cerevisiae*, *Aspergilli*

## Abstract

Plasma membrane transporters play pivotal roles in the import of nutrients, including sugars, amino acids, nucleobases, carboxylic acids, and metal ions, that surround fungal cells. The selective removal of these transporters by endocytosis is one of the most important regulatory mechanisms that ensures a rapid adaptation of cells to the changing environment (e.g., nutrient fluctuations or different stresses). At the heart of this mechanism lies a network of proteins that includes the arrestin‐related trafficking adaptors (ARTs) which link the ubiquitin ligase Rsp5 to nutrient transporters and endocytic factors. Transporter conformational changes, as well as dynamic interactions between its cytosolic termini/loops and with lipids of the plasma membrane, are also critical during the endocytic process. Here, we review the current knowledge and recent findings on the molecular mechanisms involved in nutrient transporter endocytosis, both in the budding yeast *Saccharomyces cerevisiae* and in some species of the filamentous fungus *Aspergillus*. We elaborate on the physiological importance of tightly regulated endocytosis for cellular fitness under dynamic conditions found in nature and highlight how further understanding and engineering of this process is essential to maximize titer, rate and yield (TRY)-values of engineered cell factories in industrial biotechnological processes.

## Graphical abstract

**Figure:**
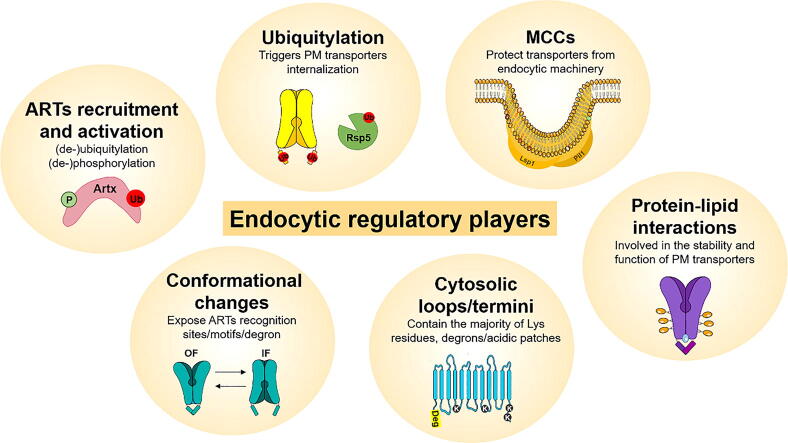

## 1 Introduction

Fungi use different sensing and signaling pathways to respond to environmental changes in nutrient availability, ensuring a sustained supply of energy, cellular growth and survival (reviewed in [1]). Cells need to adapt to fluctuations in the concentration of organic macronutrients, such as sugars, amino acids and carboxylic acids as well as essential mineral micronutrients including the metal ions copper, iron, zinc, and manganese. These substrates are mostly polar and charged and therefore their uptake is predominantly governed by the action of plasma membrane (PM) transporter systems.

**Fig. 1:**
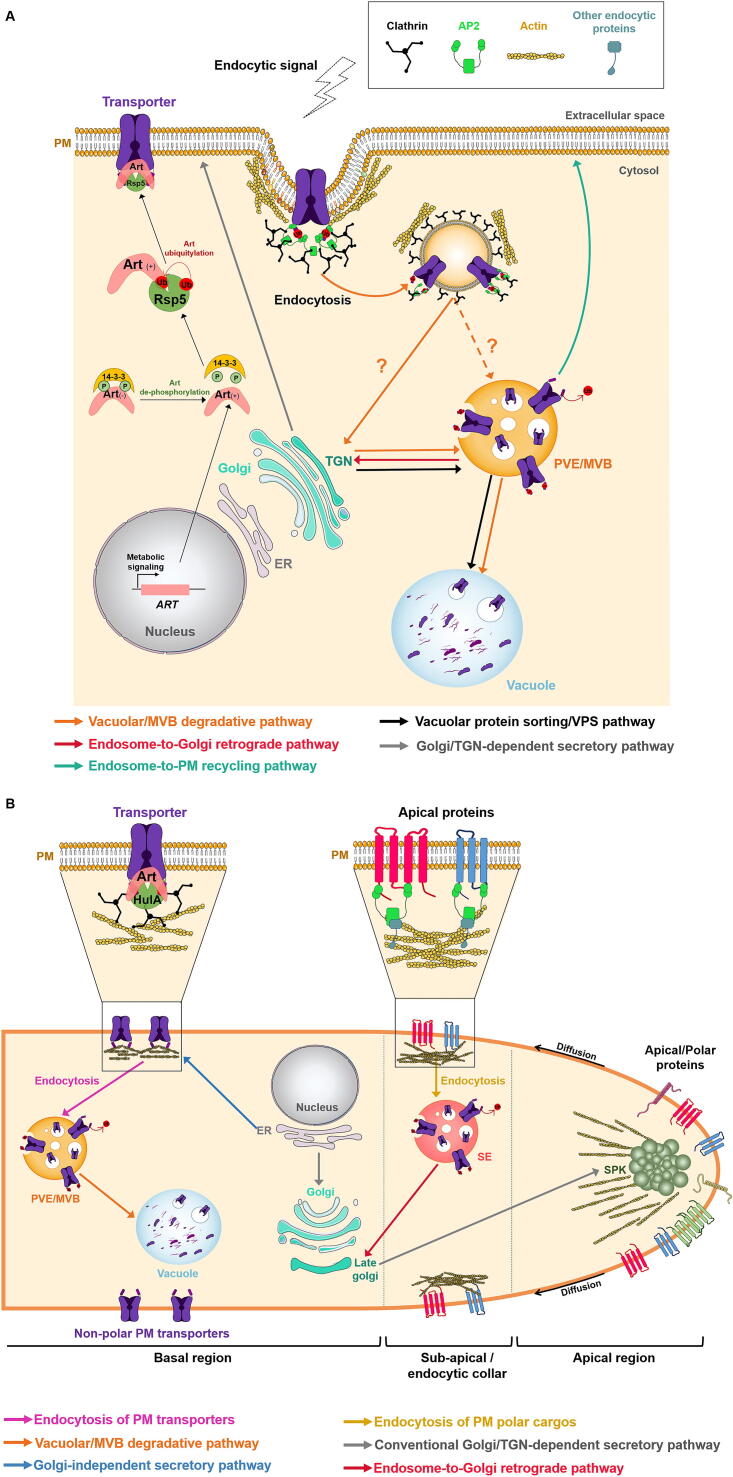
Overview of endocytosis and main trafficking pathways in budding yeast and filamentous fungi. (A) Clathrin-mediated endocytosis and main trafficking pathways of plasma membrane proteins in budding yeast (adapted from [6], [7], [8], [9], [10], [57], [58], [59], [60]). Environmental changes, stress or specific compounds (endocytic signals) can trigger PM nutrient transporter endocytosis, a process normally preceded by PM transporter ubiquitylation, mediated by Rsp5-ART complexes, and dependent on clathrin and on the AP2 complex. According to the Day et al. recent model [6], cargo proteins internalized into endocytic vesicles are sorted to the TGN (which is proposed to also serve as an early and recycling endosome). They are then delivered to the PVE/MVB, where cargo can i) be recycled back to the PM (endosome-to-PM recycling pathway); ii) be directed to the endosome-to-Golgi retrograde trafficking pathway and be secreted and recycled back to the PM, via the secretory pathway; iii) be targeted for vacuolar degradation by the vacuolar/MVB degradative pathway. It is still unclear if some endocytic vesicles can be targeted directly from the PM to the PVE/MVB. Newly-synthetized PM transporters at the ER are thought to be targeted from the Golgi and can then be sorted either to: i) the PM via the secretory pathway, or ii) to the vacuole indirectly, via vacuolar protein sorting pathway or iii) directly to the vacuole via alkaline phosphatase pathway. The latter pathway is not explored in the context of this review, so it will not be further detailed. **(B)Endocytosis and main trafficking pathways of plasma membrane transporters and polar proteins in *Aspergillus nidulans*** (adapted from [54], [55], [56]). In *A. nidulans* , there are two distinct endocytic pathways. The pathway required for the internalization of PM transporters involves their ubiquitylation by HulA^Rsp5^ -Art complexes, and depends on clathrin but not on AP2. All internalized transporters studied, so far, are degraded in the vacuole via the MVB degradative pathway. The other endocytic route, essential for polar growth, is for apical PM proteins that diffuse laterally to the sub-apical/endocytic collar (enriched in actin patches), where they are internalized by a clathrin-independent, but AP2-dependent process. Internalized vesicles are targeted to sorting endosomes (SE), then to the late Golgi/TGN, via endosome-to-Golgi retrograde pathway. From this point, AP1/clathrin coated-vesicles transport polar cargo to the so-called Spitzenkörper (SPK), from which polar proteins fuse to the PM. Additionally, two different secretory pathways were also described [53], [55]. While polar proteins follow the conventional Golgi-to-TGN dependent secretory pathway, newly synthetized transporter proteins traffic from the ER to the PM by an alternative pathway, without passing through the Golgi. ER, endoplasmic reticulum; MVB, multi vesicular bodies; PVE, pre-vacuolar endosome; PM, plasma membrane; TGN, trans-Golgi network; SE, sorting endosomes; SPK, Spitzenkörper; signals (+) and (-) represent activation and inhibition, respectively.

PM proteins are synthesized at the endoplasmic reticulum (ER) and they are targeted from the Golgi to the PM via the secretory pathway (Fig. 1). The expression/activity of these proteins at the PM is tightly regulated at the transcriptional and post-translational level, the later including endocytic downregulation and recycling. Rapid and dynamic turnover of nutrient transporters by endocytosis allows cells to quickly respond and adapt to nutrient fluctuations.

Endocytic processes have been thoroughly characterized in model fungi such as *Saccharomyces cerevisiae* or *Aspergillus nidulans* [2], [3], [4], [5]. Endocytosis is usually preceded by transporter ubiquitylation (the covalent attachment of the 76-amino-acid polypeptide ubiquitin) which signals the PM transporter for internalization. A new model of the yeast endosomal system by Day et al. [6] proposes that after internalization primary endocytic vesicles are targeted directly to the *trans*-Golgi network (TGN) and not to an early endosome, as in mammalian cells or as in the “traditional” yeast model. From the TGN, cargo is sorted to the prevacuolar endosome (PVE) [6] (Fig. 1A). The model proposes that the yeast TGN includes the organelles previously termed late Golgi and early endosome, and that the PVE has the properties of the late endosome and multi vesicular bodies (MVB). At the PVE, cargo proteins can follow different destinations: i) can be recycled back to the PM directly (endosome-to-PM recycling pathway), ii) be directed to the endosome-to-Golgi retrograde trafficking pathway and, then, be secreted, via the secretory pathway, and recycled back to the PM. Alternatively, cargo proteins can follow the vacuolar degradative pathway, traditionally referred as “MVB degradative pathway” or “MVB pathway”, which is mediated by the endosomal sorting complex required for transport (ESCRT) machinery (reviewed in [7], [8], [9], [10], Fig. 1A).

In *S. cerevisiae* and *A. nidulans*, ubiquitylation is catalyzed by a major NEDD4^1^ -like HECT^2^ E3 ubiquitin ligase, called Rsp5 or HulA, respectively [11], [12], [13]. Rsp5/HulA are highly conserved in fungi [14] and their specificity depends on a family of arrestin-related trafficking adaptors (ARTs), the so-called α-arrestins. These adaptors of Rsp5 bring the ubiquitin ligase into the vicinity of the selected PM transporter [15], [16], [17], [18], [19] (Fig. 1). ARTs also seem to promote cargo incorporation into clathrin-coated vesicles [20]. Each ART contains an N-terminal arrestin-like domain and multiple C-terminal PPxY motifs that bind to the WW domains of Rsp5, forming Rsp5-ART complexes [12], [21] that are then able to specifically ubiquitylate the PM transporter [7], [15], [22] (Fig. 1A). The arrestin binding motifs are frequently short acidic sequences (degrons) localized at the amino- (N-) and carboxylic- (C-) termini of the transporter, which interact with the basic C-terminal regions of ARTs [21], [23].

ARTs are phylogenetically conserved, from yeast to humans. In *S. cerevisiae*, 14 α-arrestins were identified: Ldb19/Art1, Ecm21/Art2, Aly2/Art3, Rod1/Art4, Art5; Aly1/Art6, Rog3/Art7, Csr2/Art8, Rim8/Art9, Art10, Bul1, Bul2, Bul3 and Spo23 [15], [16], [20], [24], [25], [26]. Filamentous fungi seem to have 7–12 predicted arrestin-like proteins [19] and, specifically, in *A. nidulans*, 10 putative ARTs were already described: CreD [17] (similar to Rod1/Art4 and Rog3/Art7) [19], PalF (homologue of yeast Rim8/Art9) [17], ApyA [19] and Arts (ArtA (similar to Art1), ArtB, ArtC, ArtD, ArtE, ArtF and ArtG [19]).

ARTs are activated and recruited in response to distinct physiological or stress signals, including nutrient excess, limitation or depletion, alkali-, heat- or hypo-osmotic shock, as well as by hydrophilic compounds and other drugs that disturb the PM structure (mostly by increasing its fluidity) or affect nutrient signaling pathways [5], [27], [28]. These adaptor proteins are therefore important regulators that connect environmental signals to the endocytosis of PM transporters, promoting cell adaptation and survival to nutrient variations and stress conditions.

ARTs are functionally redundant: a given α-arrestin can bind one or more transporters, or a particular transporter can be ubiquitylated by multiple ARTs, under distinct stress or environmental conditions [16] (see Table 1).

**Table 1:** Summary of the known mechanisms and features involved in the degradation of *S. cerevisiae* and *Aspergilli* nutrient transporters.

Transporter	Physiological substrates	Degradation signal	Ub-sites	Phospho-sites	Arrestins or Rsp5 adaptor proteins	Arrestin binding motifs	Signaling complexes (putative)	Transport activity	References
AgtA *A. nidulans*	Aspartate Glutamate	Ammonium	ND	ND	ND	ND	ND	ND	[19], [172]
AzgA *A. nidulans*	Purines: Adenine, Guanine, Hypoxanthine	Substrates or analogues	ND	ND	ArtA	ND	ND	ND	[19]
Can1 *S. cerevisiae*	Arginine, Lysine, Histidine	Arginine	K42, K45	ND	Ldb19/ Art1	70–81 aa	TORC1/Npr1	Required	[3], [15], [21], [23], [49], [178]
					Bul1/2	62–69 aa		ND	
		Cycloheximide	ND	ND	Ldb19/ Art1	Residues in the N-terminus			
		Rapamycin, Oxidative stress			ND	ND			
		Amino acids and nitrogen starvation			Ecm21/ Art2	567–575 aa	GAAC pathway	Not required	
Ctr1 *S. cerevisiae*	Copper	Excess copper	K340, K345	ND	Bul1/2	ND	ND	Not required	[21], [208]
		Amino acids and nitrogen starvation	ND		ND			ND	
Dip5 *S. cerevisiae*	Glutamic acid, Aspartic acid, Serine, Asparagine, Glutamine, Glycine, Alanine	Aspartic acid Glutamic acid	ND	T10, S11, T12, S13, S17, S18, S19, and S22	Aly1/ Art6 and Aly2/ Art3	ND	ND	ND	[156], [157], [241]
Ftr1-Fet3 *S. cerevisiae*	Iron	Excess Iron	Lys residues in either Ftr1 or Fet3	ND	ND	ND	ND	Required	[21], [49], [58], [209], [210], [242]
		Amino acid and nitrogen starvation	ND					ND	
Fur4 *S. cerevisiae*	Uracil	Uracil (extracellular and intracellular)	K38, K41	S43, S55, S56	NS	94–111 aa	ND	Required (only in Fur4-endocitosis induced by external uracil)	[16], [21], [41], [154], [178], [190]
		H_2_O_2_ Heat shock		ND	ND			ND	
		Rapamycin, Heat shock, Oxidative stress, Alcoholic stress	ND			ND			
FurE *A. nidulans*	Uracil, Allantoin, Uric acid	Uric acid, Allantoin, Uracil (less efficiently), Ammonium	K521, K522	ND	ND	501–503 aa	ND	Required	[5], [38], [39], [188]
Gap1 *S. cerevisiae*	Various amino acids	Extracellular ammonium or amino acids	K9, K16	ND	Bul1/2	ND	TORC1/Npr1	Required	[20], [23], [30], [163], [178], [243]
		Intracellular ammonium or amino acids				20–35 aa		Not required	
		Rapamycin, Heat shock, Oxidative stress, Alcoholic stress			Bul1/2, Aly2/ Art3 and Aly1/ Art6	Residues in the C-terminus		ND	
Hxt1 *S. cerevisiae*	Glucose, Fructose, Mannose	Low Glucose	K12, K39	ND	ND	ND	Ras/cAMP-PKA	ND	[74], [102], [107]
		Rapamycin	ND				Ras/cAMP-PKA TORC1/Npr1		
		2-deoxyglucose			Rod1/ Art4	ND	ND Snf1/AMPK		
Hxt2 *S. cerevisiae*	Glucose, Fructose, Mannose, Pentose	High Glucose	ND	ND	ND	ND	Snf1/AMPK PKA	ND	[16], [36], [75]
		Low glucose			Crs2/ Art8				
Hxt3 *S. cerevisiae*	Glucose, Fructose, Mannose	Low Glucose	ND	ND	Crs2/ Art8	ND	Ras/cAMP-PKA Rim15	ND	[76], [102]
		2-deoxyglucose			Rod1/ Art4 and Rog3/ Art7	ND	ND Snf1/AMPK		
Hxt4 *S. cerevisiae*	Glucose, Fructose, Mannose, Pentose	Low glucose	ND	ND	Crs2 (Art8)	ND	Snf1/AMPK PKA	ND	[36]
Hxt5 *S. cerevisiae*	Glucose, Fructose, Mannose	Low growth rate	ND	ND	ND	ND	Ubiquitin-independent	ND	[77]
Hxt6 *S. cerevisiae*	Glucose, Fructose, Mannose	High Glucose	ND	ND	Rod1/ Art4	ND	Snf1/AMPK	ND	[16], [31], [36], [78], [96]
		Low glucose			Crs2/ Art8		Snf1/AMPK PKA		
		Cycloheximide			Crs2/ Art8		ND		
Hxt7 *S. cerevisiae*	Glucose, Fructose, Mannose	High Glucose	ND	ND	ND	ND	Ras/cAMP-PKA	ND	[36], [76], [78], [79], [80]
		Nitrogen starvation, Rapamycin			ND		TORC1 Ras2 Rim15		
		Low glucose			Crs2/ Art8		Snf1/AMPK PKA		
Jen1 *S. cerevisiae*	Lactate, Pyruvate, Acetate, Propionate	Glucose	K63, K338, K599, K607	ND	Rod1/ Art4	612–614 aa	TORC1/Npr1	Not required	[24], [47], [123], [125], [126]
		Alkali stress, Cycloheximide	ND		Bul1	ND		Required	
		Rapamycin						Not required	
Lyp1 *S. cerevisiae*	Lysine	Lysine	ND	ND	Ldb19/ Art1	ND	ND	ND	[14], [15], [22], [114]
		Cycloheximide			Ecm21/ Art2	Residues in the N-terminus			
		Rapamycin, Heat shock, Oxidative stress, Alcoholic stress			ND	ND			
		Amino acids and nitrogen starvation			Ecm21/ Art2	588–598 aa (predicted)	GAAC pathway	Not required	
Mal61 *S. cerevisiae*	Maltose, Turanose	Glucose	ND	ND	ND	48–79 aa	Snf1/AMPK Snf3/Rgt2	ND	[84], [244], [245]
MalP *A. oryzae*	Maltose	Glucose	ND	ND	CreD	ND	ND	ND	[111], [239]
		Mannose			ND				
		2-deoxyglucose							
Mup1 *S. cerevisiae*	Methionine, Cysteine	Methionine	K27, K28	ND	Ldb19/ Art1	41–55 aa	ND	Required	[16], [21], [46], [154], [246]
		Amino acids and nitrogen starvation	K567, K572	T552, T560	Ecm21/ Art2	549–555 aa	GAAC pathway	Not required	
PrnB *A. nidulans*	Proline	Ammonium	ND	ND	ArtA	ND	ND	ND	[19], [173]
Smf1 *S. cerevisiae*	Di-valent and tri-valent metals: Manganese, Iron, Copper, Cadmium, Cobalt, Nickel,	Physiological manganese	ND	Residues in the N-terminus	Bsd2, Tre1 and Tre2	ND	ND	Required	[60], [106], [214], [230], [232]
		Cadmium	K33, K34	ND	Ecm21/ Art2, Crs2/ Art8			Not required	
		Excess manganese	ND		ND				
		Amino acid and nitrogen starvation	ND					ND	
Tat2 *S. cerevisiae*	Tryptophan, Tyrosine	Tryptophan	ND	ND	Ldb19/ Art1 and Bul1	ND	ND	ND	[15], [16], [21]
		Cycloheximide			Ecm21/ Art2 and Crs2/ Art8				
		Amino acids and nitrogen starvation	ND	ND	Ecm21/ Art2	561–570 aa (predicted)	GAAC pathway	Not required	
UapA *A. nidulans*	Uric acid, Xanthine	Purines (xanthine, uric acid)	K572	ND	ArtA	545–547 aa	ND	Required	[18], [19]
		Primary nitrogen source (ammonium or glutamine)						Not required	
UapC *A. nidulans* *A. oryzae*	Purines: Uric acid, Xanthine, Hypoxanthine, Adenine, Guanine	Ammonium	ND	ND	ND	ND	ND	ND	[197], [198], [199]
Zrt1 *S. cerevisiae*	Zinc	Excess Zinc	K195	ND	ND	ND	ND	ND	[211], [212], [213], [242]
		Cadmium							
		Cobalt	ND						
		Amino acids and nitrogen starvation	ND		ND			ND	

ND – Not Determined; NS – Non Specific; aa - amino acids.

**Fig. 2:**
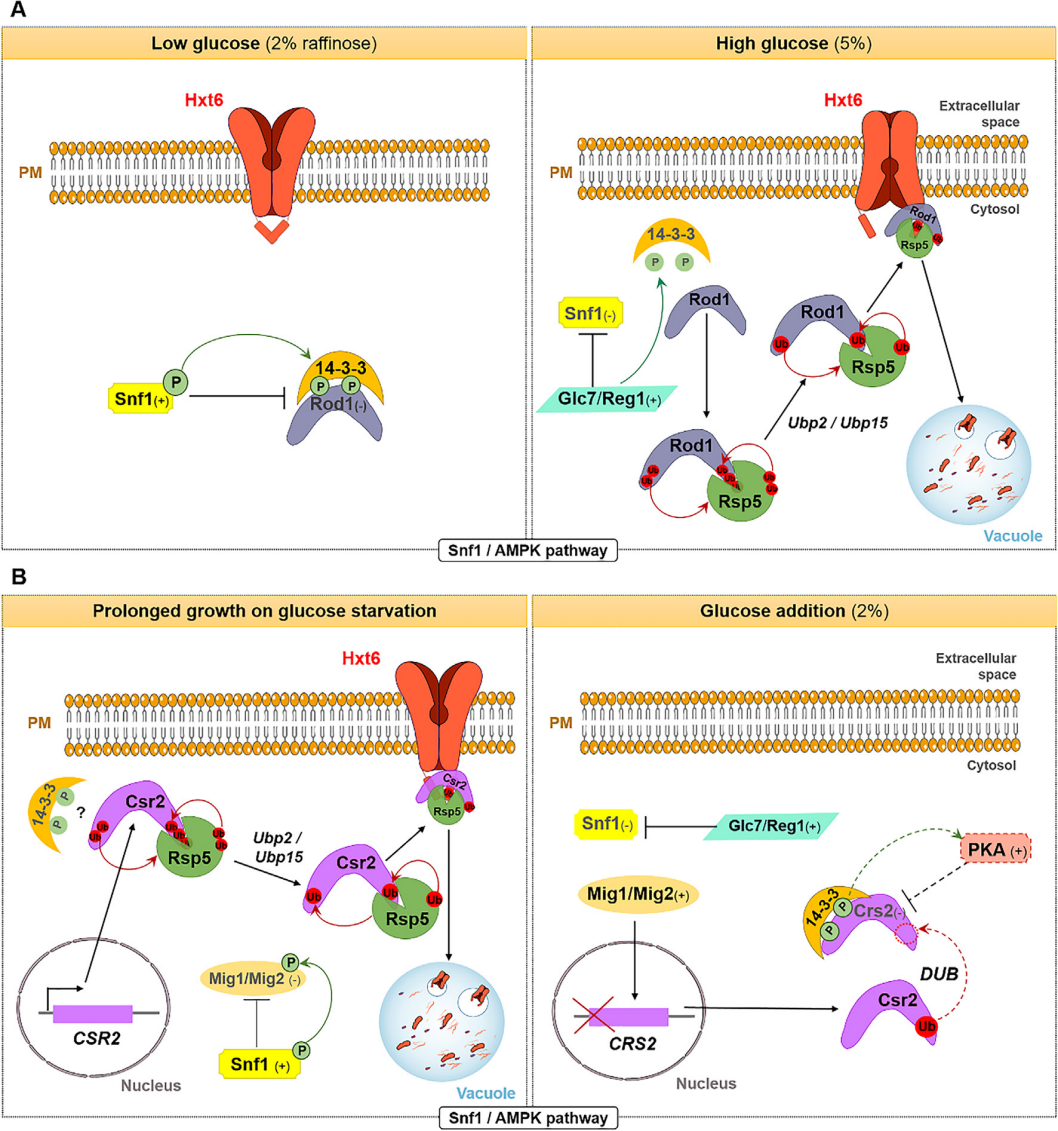
Endocytosis of Hxt6 induced by distinct signals. (A) Hxt6 degradation induced by glucose addition to cells grown on glucose limiting conditions is controlled by the Snf1/AMPK pathway. In glucose-limiting conditions, Hxt6 and Rod1/Art4 are inactive. Glucose addition triggers PP1 phosphatase (Glc7/Reg1) activation, resulting in Snf1 dephosphorylation and the release of Rod1 from 14 to 3-3 proteins. Rod1 is then continuously ubiquitylated by Rsp5 [16], [31], but Ubp2 and Ubp15 lead to Rod1 deubiquitylation, preventing its subsequent proteasomal degradation [101]. Rod1-Rsp5 complexes promote the transporter ubiquitylation and its subsequently degradation in the vacuole [31], [106]. (B) Hxt6 internalization in response to prolonged growth in glucose starvation conditions. During prolonged growth in glucose starvation conditions (e.g., growth in lactic acid, 0.5%, w/v, pH 5.0, for 24 h*),* Snf1 kinase is active and inhibits Mig1/Mig2 repressors by phosphorylation, preventing them from binding to *CRS2* promoter and resulting in *CRS2* derepression. The increase in *CRS2* transcription elevates Crs2 protein levels and the consequent formation of Rsp5-Crs2 complexes. This ultimately leads to Hxt6 ubiquitylation and degradation through the MVB pathway. In contrast, upon a pulse of glucose (2%, w/v), PP1 phosphatase (Glc7/Reg1) dephosphorylates and inactivates Snf1, which can no longer phospho-inhibit Mig1/Mig2, resulting in *CRS2* repression. At the posttranslational level, Crs2 protein is inactivated by phosphorylation, possibly by PKA kinase, leading to its association with 14–3-3 proteins and its deubiquitylation [36]. Ub, ubiquitylation; P, phosphorylation; PM, plasma membrane. Dashed lines represent predicted regulation and signals (+) and (-) represent activation and inhibition, respectively.

The regulation of ARTs depends on posttranslational modifications such as phosphorylation and ubiquitylation and involves distinct signaling pathways (reviewed in [28], [29]). While the phosphorylation of ARTs leads to their inactivation, dephosphorylation seems to facilitate α-arrestins mediated endocytosis. The phosphorylation and dephosphorylation of ARTs C-terminal residues is catalysed by protein kinases and phosphatases, respectively, whereas their ubiquitylation is mediated by the Rsp5/HulA ligase. In *S. cerevisiae*, protein kinases that were found to directly phosphorylate (inactivate) ARTs are Snf1 (the yeast homologue to the human AMP-activated protein kinase - AMPK), Npr1 (nitrogen permease reactivator 1), Pho85, Yck1/Yck2 (yeast casein kinase 1/2) and Ypk1 (a serine-threonine protein kinase). Examples of protein phosphatases that directly or indirectly control the activity of ARTs include calcineurin, Glc7 (catalytic subunit of type 1 protein phosphatase (PP1)) and Sit4 (a serine-threonine phosphatase) (reviewed in [28]). When phosphorylated, some α-arrestins were reported to bind Bmh1 and Bmh2 protein isoforms [30], [31], [32] of the 14-3-3 protein family [33], [34]. This class of eukaryotic conserved proteins play important regulatory roles in several physiological processes, including signal transduction, metabolism, regulation of cell cycle, stress response and protein trafficking (reviewed in [35]). Despite ARTs posttranslational regulation, these adaptor proteins are also subjected to transcriptional control by nutrient sensing pathways [21], [36] (see following sections and Fig. 1A; 2B; 5B).

In addition to ARTs regulation, the cytosolic N- and C-termini of the nutrient transporter also play an important role in endocytosis (reviewed in [37]). These termini contain the majority of Lys residues and acidic motifs necessary for transporter ubiquitylation and, in some cases, the interaction between the N- and C-termini appears to control the accessibility of these motifs to the ubiquitylation machinery (Rsp5 and α-arrestins) [5], [23], [37], [38], [39]. Indeed, the access of ARTs to specific residues/motifs of PM proteins often requires conformational changes of the transporters, which are induced by the binding of the substrate to the transporter [38], [39], [40], [41], [42], [43], [44]. This mechanism, named substrate/activity-dependent endocytosis, operates in nutrient transporters of different fungi [18], [38], [41], [45], [46], [47], [48]. In contrast, substrate/activity-independent endocytosis does not rely on transporter structural changes induced during the substrate import cycle and it is normally induced by nutrient limitation and/or starvation conditions [21], [49].

In *Aspergillus* species, similar to *S. cerevisiae,* endocytosis also operates for the downregulation of PM nutrient transporters in response to different environmental or stress signals. However, endocytosis is also essential for the formation of filaments (hyphae) via apical extension and maintenance of polar growth [50], [51], [52]. This is reflected in the fact that several mutations affecting endocytosis are lethal in contrast to yeasts [4].

The continuous process of internalization and recycling of apical proteins ensures the maintenance of the polar growth of the tip [53]. Nevertheless, the endocytic mechanism for polar membrane cargoes close to the tip, which in most cases are required for PM and cell wall synthesis, is distinct from the endocytic process of non-polar proteins such as nutrient transporters, localized at the basal region (reviewed in [54]). Specifically, the internalization of nutrient transporters is AP2 independent, despite being clathrin-dependent, while the endocytosis of polarly localized (apical) membrane proteins are AP2 dependent but clathrin-independent [55] (Fig. 1B). In *A. nidulans*, apical PM proteins and nutrient PM transporters were also shown to follow different secretory pathways. Polar cargo traffic to the apical membrane via the conventional Golgi/TGN-dependent secretory/trafficking route, in contrast to *de novo* synthetized nutrient transporters that traffic to the PM via a recently discovered Golgi-independent route, apparently directly from the ER [50], [51], [54], [56] (Fig. 1B).

In the different sections of this review, we highlight the main regulatory circuits required for the dynamic turnover of specific transporters, induced by distinct signals. We will focus on a set of important macro- (carbon (C) and nitrogen (N)) and micro- (copper, iron, zinc and manganese) nutrient transporters known to be regulated by endocytosis in the budding yeast *S. cerevisiae*. We also highlight what is known about these processes in two different species of the filamentous fungus *Aspergillus* (*A. nidulans* and *A. oryzae)*. Furthermore, we underline the physiological importance of these processes for adaptation to dynamic cellular environments and illustrate how engineering of these systems has the potential to improve titer, rate and yield (TRY) in industrial biotechnological processes.

## 2 Endocytosis of sugar transporters

Yeasts use a wide variety of sugars such as hexoses as primary C and energy sources. In *S. cerevisiae*, transport of these hexoses is mediated by some members of the Hexose transporter family (Hxt), composed of 20 PM proteins (Hxt1 to Hxt17, Gal2 galactose transporter and Snf3 and Rgt2 glucose sensors) [1], [61], [62], [63], [64], [65], [66], [67], [68]. These proteins belong to the major facilitator superfamily (MFS) and are composed by a cytosolic N- and C- termini, which vary in length, and 12 transmembrane segments (TMSs) organized into two discretely folded domains connect by an intracellular hydrophilic loop [69], [70], [71]. All members, except the sensors and Hxt12, are able to transport glucose, fructose and mannose [62], [65], but the transporters Hxt1-4 and Hxt6-7 seem to play a major role, under most physiological conditions [62], [72]. Hxt transporters were reported to operate by a facilitated diffusion mechanism, in a highly regulated manner, with different affinities for glucose, fructose and mannose [73]. In *S. cerevisiae* cells, glucose is sensed (via glucose sensors Snf3 and Rgt2) over a wide range of concentrations and acts as the most important regulator, affecting the expression of these transporters at both transcriptional and post-transcriptional levels (reviewed in [71]). Besides glucose, other environmental signals can also regulate these transporters by affecting their endocytosis. The sugar transporters known to be regulated by endocytosis in *S. cerevisiae* include the hexose transporters Hxt1 [74], Hxt2 [75], Hxt3 [76], Hxt4 [36], Hxt5 [77], Hxt6 [31], [78] and Hxt7 [78], [79], [80], the galactose transporter Gal2 [81], [82], and the maltose transporters Mal11, Mal21 and Mal61 [83], [84], [85] (Table 1).

The genome of *Aspergilli* contains many genes coding for proteins of the MFS [86]. In some *Aspergillus* species, the genes encoding proteins involved in sugar transport were already identified. For instance, *A. oryzae* and *A. niger* contain 127 [87] and 86 [88] putative sugar transporter genes, respectively. However, only a small number of these transporters has been functionally characterized [86], [89], [90], [91], [92], [93], [94]. These include the glucose transporters HxtA [89], HxtB-C [92] and MstE [91], the xylose transporter XtrD and the cellobiose transporter CltA [86] from *A. nidulans*; the MstA sugar proton symporter [90] and the cellodextrin transporter CtA [94] from *A. niger*, and, finally, the maltose transporter Malp (Mal61 *S. cerevisiae* homologue) from *A. oryzae* [95]. In contrast to *S. cerevisiae*, endocytosis of sugar transporters in *Aspergilli* remains poorly studied.

### 2.1 Endocytosis of sugar transporters triggered by excess of substrate

The high-affinity glucose transporters Hxt6 and Hxt7 [16], [31], [78], [80], [96] and the medium-affinity glucose transporter Hxt2 [75] are rapidly internalized and triggered for vacuolar degradation in response to high external glucose concentrations (5%, w/v). Control of the glycolytic flux is largely dependent on sugar transport [97], which has been described to be limited by the PM space required for incorporation of the transport proteins [98], [99]. Consequently, competition for the limited PM space between sugar transporters with different activities [100] impacts overall transport rates which makes removal of inefficient transporters in dynamic culture conditions essential to optimize strain performance. Therefore, endocytosis of medium and high affinity transporters triggered by substrate excess could be essential to liberate additional PM space for low-affinity, high-capacity transporters, which are able to catalyze the highest rates of glucose transport under these conditions.

Specifically, under glucose-limited conditions (e.g., cells growing in raffinose as sole C source^3^), Hxt6 is localized at the cell surface, Snf1 (the yeast homologue of human AMP kinase) is active, promoting Rod1/Art4 phosphorylation and, consequently, its inactivation. Rod1 can also bind to Bmh1/2 14-3-3 proteins, stabilizing the inactive-phosphorylated form of ART adaptors. Addition of glucose activates the type 1 protein phosphatase (PP1), composed of Glc7 catalytic subunit and Reg1 regulatory subunit, which promotes dephosphorylation and inactivation of Snf1. This leads to Rod1 activation through its dephosphorylation and release from 14 to 3-3 proteins. Once free, Rod1 is continuously ubiquitylated by Rsp5 E3 Ub ligase [16], [31], but the deubiquitinating enzymes (DUBs) Ubp2 and Ubp15 promote Rod1 deubiquitylation and, consequently, prevent its hyperubiquitylation and subsequent proteasomal degradation [101]. The formation of Rod1-Rsp5 complexes culminates in Hxt6 ubiquitylation and its subsequent degradation in the vacuole [16], [31] (Fig. 2A). A general model shared by most of the ART adaptors was proposed, in which the DUBs Ubp2 and Ubp15 affect endocytic trafficking by regulating ARTs stability [101].

### 2.2 Endocytosis of sugar transporters triggered by substrate depletion or starvation

The low-affinity, high-capacity transporters Hxt1 and Hxt3 are expressed when glucose is abundant (2%, w/v). When glucose becomes depleted, these transporters are targeted for endocytosis, which requires their ubiquitylation by Rsp5 and the inactivation of the Ras/cAMP-PKA glucose signaling pathway [74], [76] (Table 1). Hxt1 ubiquitylation caused by substrate depletion requires its N-terminus, and occurs at Lys12 and Lys39 residues [74]. Glucose starvation-induced turnover of Hxt3 is dependent on Csr2/Art8 adaptor and requires Rim15, which is a downstream effector kinase of the Ras/cAMP/PKA pathway [76]. Addition of 2-deoxyglucose (2DG), which mimics glucose starvation, also triggers Hxt1 and Hxt3 endocytosis [102]. This glucose analogue induces formation and intracellular accumulation of 2-deoxy-d-glucose-6-phosphate (2-DG6P), which, in turn, triggers hexokinase inhibition and, consequently, inhibition of glycolysis [103]. However, 2DG-mediated endocytosis of Hxt3 requires both Rod1 and Rog3/Art7 arrestins, whereas Rod1 is the sole arrestin responsible for Hxt1 2DG-dependent endocytic degradation. The loss of Snf1 also results in the downregulation and vacuolar degradation of these transporters [102]. Remarkably, Rod1 suffers some degree of inactivation (phosphorylation) by the action of Snf1 [102]. Considering that 2DG mimics glucose starvation, the inhibitory action of this kinase may allow some glucose import into the cell by counteracting the Rod1- and Rog3-induced internalization of Hxt1 and Hxt3. However, when compared to the activity of cells experiencing glucose starvation, Snf1 activity is modest [102].

The endocytosis of medium- to high-affinity glucose transporters, including Hxt2, Hxt4, Hxt6 and Hxt7, was shown to be triggered when cells are grown for a prolonged time in medium containing lactic acid (0.5%, w/v, pH 5.0), as the sole C source, a condition that, the authors claim, also mimics glucose starvation [36]. These conditions were previously reported to trigger the downregulation of *S. cerevisiae* Jen1 lactate transporter, a mechanism which, in this case, was found to be associated with alkali stress [47]. However, in contrast to what was observed for Jen1, prolonged growth in ethanol and even C starvation, also induced the degradation of the above-mentioned glucose transporters, suggesting a different mechanism of regulation (see Fig. 2B, showing the regulated endocytosis of Hxt6 as an example). The internalization of these transporters is triggered in an ubiquitin- and Rsp5-dependent manner, but only Hxt6 and Hxt7 degradation requires the Crs2 arrestin [36]. In the absence of glucose, *CRS2* is transcriptionally induced, as Mig1 and Mig2 repressors are inhibited by AMPK kinase Snf1. Upon protein synthesis, Crs2 becomes activated by ubiquitylation and acts as an adaptor protein for Rsp5 to mediate cargo ubiquitylation. In contrast, the presence of glucose inhibits the transcription of *CRS2* and promotes Crs2 phosphorylation, its association with Bmh1/Bmh2 (14-3-3) proteins and its deubiquitylation. Importantly, PKA kinase appears to contribute to Crs2 inactivation, revealing an unexpected crosstalk between Snf1/AMPK and PKA pathways [36] (Fig. 2B).

Tightly coordinated sugar transporter expression and endocytosis could have evolved in environments where both mono- and disaccharides are present to prevent futile cycling of these nutrients across the PM. For example, disaccharides such as maltose are transported by proton (H^+^)-coupled α‐glucoside transporters belonging to the maltose permease subfamily [104]. By coupling maltose transport to the proton-motive-force, these transporters enable maltose accumulation inside the cells, where subsequent hydrolysis releases two molecules of glucose for further conversion. In the presence of Hxt transporters, these glucose monomers would be transported out of the cell, down the concentration gradient. Since maltose transporters Agt1, Mph2 and Mph3 can also catalyze H^+^-coupled transport of glucose [65] glucose release by Hxt transporters would be followed by H^+^-coupled re-uptake via these α‐glucoside transporters and ultimately result in a net translocation of H^+^ into the cell [104]. Proton export via the *S. cerevisiae* H^+^-ATPase Pma1 results in a net energetic expense of ATP and this cycle would ultimately deplete cellular energy [105].

### 2.3 Endocytosis of sugar transporters triggered by different physiological inputs

Some sugar transporters are also internalized in response to different types of stress (Table 1). Hxt1 is ubiquitylated, endocytosed and degraded in response to rapamycin (inhibitor of TORC1) [107]. Also, Hxt7 is internalized in response to rapamycin or N starvation, in a mechanism involving TORC1 and Ras2 inactivation and the requirement of Rim15 kinase, a common downstream effector of both PKA and TOR pathways [76], [79]. Cycloheximide (inhibitor of protein synthesis, activator of TORC1) has also been reported to trigger the degradation of Hxt6 in a Crs2 dependent manner [16].

The expression of the medium-affinity glucose transporter Hxt5 at the PM is induced when cells experience low growth rates, including in glucose-grown cells at stationary phase, in cells cultivated under poor non-fermentable C sources, and when temperature or osmolarity increase [64], [108], [109]. In *S. cerevisiae*, expression of this transporter occurs before and during the diauxic shift, when metabolic changes prepare the switch to ethanol as C source [108], [110]. When cells are exposed to glucose again, Hxt5 is transiently phosphorylated in its serine residues, internalized and degraded in the vacuole. This coordinated Hxt5 expression and retention in the PM under glucose-depleted conditions hints towards a central role of this transporter in keeping glucose-starved cells prepared for future glucose exposure. Although internalization and degradation of Hxt5p occur in a ubiquitin-independent manner via the endocytic pathway [77], additional studies are required to further investigate the signaling events involved in growth-rate-dependent Hxt5 turnover.

In *Aspergillus* species the endocytic regulation of the maltose transporter MalP from *A. oryzae* has already been reported. This PM transporter is downregulated when amylolytic enzyme production is repressed, which occurs after addition of glucose, mannose or 2-deoxyglucose [111]. The internalization of MalP, triggered by glucose addition, requires its ubiquitylation by the HulA^Rsp5^ E3 ubiquitin ligase and involves the arrestin-like protein CreD (homologue of yeast Rod1). However, in this case, the phosphorylation state of CreD does not seem to affect transporter endocytosis [111].

## 3 Endocytosis of monocarboxylate transporters

Two distinct transport systems for monocarboxylates have been reported in *S. cerevisiae*: Ato1/Ady2 [112], [113], and Jen1 [114], [115], belonging to distinct evolutionary families of transporter proteins.

Ato1 (acetate transport ortholog 1), recently reclassified [113], belongs to the acetate uptake transporter (AceTr) family and it is responsible for the uptake of acetate, propionate, formate and lactate [116]. The endocytic turnover of this transporter has been poorly investigated.

Jen1 is a monocarboxylate/proton symporter that belongs to the MFS superfamily, specifically to sialate:H^+^ symporter (SHS) family [117], [118]. It is able to transport lactate, pyruvate, acetate and propionate [114], as well as selenite [119] and it has been extensively studied at a biochemical and molecular levels [24], [47], [120], [121], [122], [123], [124], [125], [126]. The crystal structure of Jen1 has not been solved yet, but based on predicted topology studies, it contains 12 putative TMSs and a cytosolic N- and C-termini. A mutational study has also shown that a conserved motif ^379^NXX[S/T]HX[S/T]QDXXXT^391^, located in the TMS-7, seems to be involved both in transport capacity and in substrate affinity [120].

Jen1 transporter is endocytosed and degraded in the vacuole in the presence of rich C sources, such as glucose [122]. The low maximum specific growth rate of *S. cerevisiae* on lactic acid [127], indicates that this organism is not well adapted for growth on this C source alone. Its preference for glucose could be related to a lower energetic yield from complete oxidative dissimilation of lactic acid (7 ATP, including 1 ATP from substrate level phosphorylation) compared to a carbon-equivalent amount of glucose (8 ATP, including 1 ATP from substrate level phosphorylation) assuming a P/O ratio of 1 [128] in combination with a poor respiratory capacity [129], [130]. Therefore, internalization of Jen1 after addition of glucose appears to result in an energetic benefit for yeast cells.

**Fig. 3:**
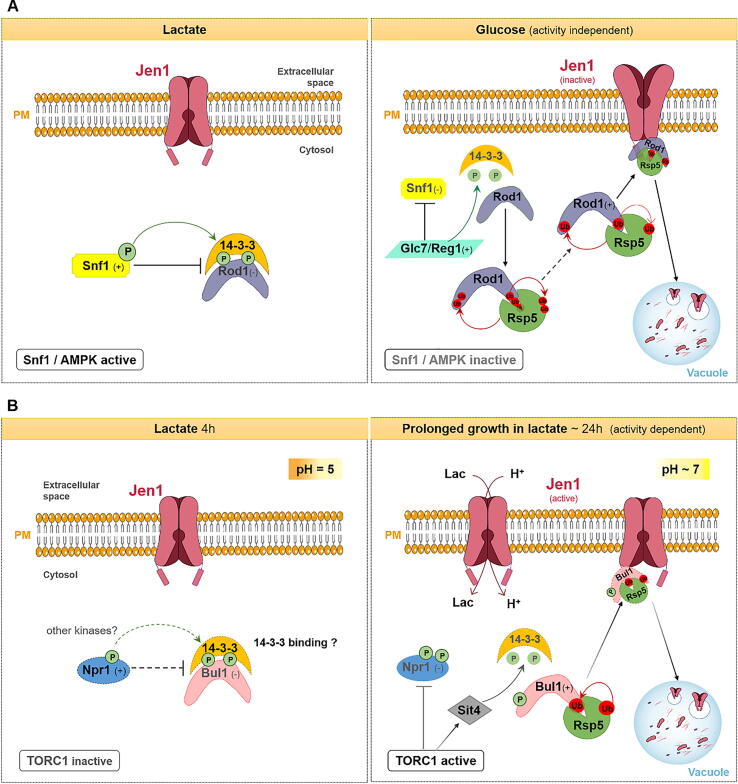
Schematic representation of Jen1 endocytosis in response to distinct signals. (A) Glucose-induced downregulation of the monocarboxylate Jen1 transporter. In the presence of lactate, Jen1 is localized at the PM and Rod1/Art4 is inactive. Upon glucose addition, Glc7/Reg1 dephosphorylates Snf1 and Rod1. Rod1 is released from a phospho-dependent binding with 14–3-3 proteins and can, then, bind Rsp5. This results in Jen1 ubiquitylation, internalization and degradation via the MVB pathway [24], [122], [123]. Ubp2 and Ubp15 seem to control Rod1 activity by managing the level of its ubiquitylation [101]. (B) Alkali stress induced internalization of Jen1 transporter. In cells induced in lactate, for 4 h, Jen1 is localized at the PM. The prolonged growth (24 h) in lactate results in the alkalinization of the extracellular medium and leads to Jen1 internalization and degradation. This mechanism depends on an active Jen1 transporter and relies on TORC1 pathway. The model proposes that activated TORC1 leads to the inactivation of Npr1 kinase, and the activation of Sit4 phosphatase. This results in Bul1 activation and consequently Jen1 Rsp5-ubiquitylation and subsequent vacuolar degradation [47]. PM, plasma membrane; Lac, lactate; Ub, ubiquitylation; P, phosphorylation; H^+^, proton. Dashed lines represent predicted regulation and signals (+) and (−) represent activation and inhibition, respectively.

Briefly, in the presence of lactate (0.5% (v/v), pH 5.0), as a sole C source, Jen1 is localized at the cell surface and Rod1/Art4 is phosphorylated (inactive) and bound to 14–3-3 proteins by the action of Snf1 kinase (yeast homologue of AMPK kinase). Upon glucose addition, the phosphatase complex Glc7/Reg1 dephosphorylates Snf1 and Rod1. While Snf1 becomes inactive, de-phosphorylation of Rod1 releases it from the phosphor-dependent binding with 14-3-3 proteins, allowing its ubiquitylation by the E3 ligase Rsp5 [24], [122], [123]. Finally, the complex Rod1-Rsp5 recognizes a specific motif/degron within the C-terminus of Jen1 (612-614 aa), triggering Jen1 internalization and degradation [126]. This substrate-independent endocytic pathway does not require an active Jen1 transporter [47]. Also, cells lacking Ubp2 and Ubp15 showed an impairment in glucose-induced Jen1 internalization, suggesting the involvement of these DUBs in controlling Rod1 activity and, consequently, in Jen1 traffic [101] (Fig. 3A).

Glucose induced endocytosis of Jen1 also seems to involve the Yck1 kinase, directly or indirectly [123]. Moreover, the replacement of all cytosolic lysine (K) residues of the transporter by arginine is sufficient to block Jen1 internalization [131]. While K9 (at Jen1 N-terminus) and K338 (at Jen1 cytosolic loop), were shown to be important, but not essential, for glucose-elicited Jen1 endocytic turnover [123], the lysine residues K599 and K607, localized at Jen1 C-terminus, seem critical for this process [126]. Besides the role of Rod1 for the initial stages of Jen1 internalization, this ART also seems to operate later at the TGN to promote Jen1 vacuolar sorting and degradation in the presence of glucose or to recycle Jen1 back to the PM, when glucose is removed [131], [132] (see VPS and recycling pathways in Fig. 1A). Moreover, Bul1 was also reported to be involved in glucose-elicited endocytosis of Jen1 [125], suggesting that it can also be a target for Snf1 kinase.

The endocytosis of Jen1 can also be triggered by other signals, such as the addition of rapamycin, cycloheximide or alkali stress, in a process involving Bul1 arrestin, but independent of Rod1/Art4 [47]. The authors of this work proposed a model for alkali-induced internalization of Jen1 (Fig. 3B): prolonged growth (24 h) in lactic acid (0.5% (v/v), pH 5.0), as sole C source, triggers alkalinization of the external medium (an increase on extracellular pH from 5 to 7), which somehow stimulates the TORC1 pathway. TORC1, in turn, promotes hyper-phosphorylation (inactivation) of Npr1 kinase and activation of Sit4. This phosphatase dephosphorylates Bul1 arrestin, possibly releasing it from a phospho-dependent binding with 14–3-3 proteins. Dephosphorylated Bul1 is then ubiquitylated by Rsp5 ubiquitin ligase and, finally, the complex Bul1-Rsp5 triggers Jen1 ubiquitylation, probably by binding at the N-terminus, resulting in its internalization and vacuolar degradation. Interestingly, while alkaline-induced Jen1 internalization requires a functional and active Jen1 transporter, internalization induced by glucose is transport-independent (Fig. 3; Table 1) [47]. These findings suggest that Jen1 efficient turnover triggered by alkali stress depends on Jen1 conformational changes, associated with monocarboxylate/H^+^ symport.

In alkali- and cycloheximide-induced Jen1 endocytosis, the requirement of TORC1 is supported by several lines of evidence by Talaia and co-workers [47]. Besides the action of TORC1 effectors (Npr1 and Sit4), the deletion of Tco89, a TORC1 subunit, causes an endocytic dysregulation of Jen1. The involvement of TORC1 in alkali stress induced endocytosis of Jen1 is further reinforced by the observation that the replacement of ammonium with proline, as sole nitrogen source, in the external growth medium, reduces Jen1 endocytic turnover. In the case of rapamycin-induced endocytosis, the requirement of TORC1 is supported by tor1/tor2 mutant́s phenotypes [47].

It is not completely clear how TORC1 complex stimulates Bul1-mediated endocytosis, in response to physiological signals, although some insight has been gained, over the past few years, into this process. Despite observed similarities between Bul1-dependent endocytosis of Gap1 and Jen1, for the latter, Bul1 remained partly phosphorylated in the absence of Npr1, in the tested physiological conditions (addition of cycloheximide and prolonged growth in lactate) [47]. Whether Npr1 kinase activity is directly or indirectly required for Jen1 endocytic turnover remains elusive. The potential involvement of other kinases, like Snf1, on Bul1 phosphorylation may reveal unexpected synergies between distinct nutrient signaling cascades. In a similar fashion, Crs2 seems to rely on two distinct signaling pathways to regulate Hxt6 endocytosis [67]. It is still enigmatic how Jen1 transporter activity leads to extracellular alkalinization, after prolonged growth in lactic acid medium, as sole C source, which, in turn, is a signal for Jen1 endocytosis. The acidic nature of Jen1 substrates, monocarboxylates and protons, used as sole C source, suggests that substrate uptake via Jen1 causes extracellular alkalinization; however, microbial cells have developed intricate mechanisms to respond to alkali pH stress, which involves the modulation of several signaling pathways and the impairment on the uptake of innumerous nutrients (reviewed in [133]). Therefore, more studies are required to deeply understand the molecular mechanisms behind Jen1 internalization induced by alkali stress.

In filamentous fungi, little is known about carboxylic acid transporters regulation. In *A. nidulans*, homologs of *S. cerevisiae* Ato1 (AcpA and AcpB) and Jen1 (JenA and JenB) transporters were identified [134]. However, their endocytic regulation has not been investigated yet.

## 4 Endocytosis of amino acid transporters

Amino acids (AAs) are a major N and/or C source for fungi. *S. cerevisiae* cells harbor many fungal AA transporters (fAATs) that differ according to substrate specificity, cellular location, regulation and protein fold [135]. fAATs are grouped into two major superfamilies: MFS and the APC (amino acid-polyamine-organocation) superfamilies [136]. However, the majority of PM fAATs belong to the APC superfamily, which is subdivided into three major families: the YAT (Yeast Amino acid Transporter) family, the LAT (L-type Amino acid Transporter) family and the ACT (Amino Acid/Choline Transporter) family [137], [138], [139]. Members of YAT family contain 12 TMSs and share a common structural LeuT fold formed by TMs 1-10 [140]. So far, no YAT structure has been determined and the structure of the transporters belonging to this family is based on other solved transporter structures sharing the LeuT conformation, namely the arginine/agmatine AdiC transporter from *Escherichia coli* [140], [141].

In the yeast *S. cerevisiae*, 22 PM AA transporters, with known function, were already identified (reviewed in [140]). These permeases, similar to sugar transporters, are also regulated post-transcriptionally in response to excess of substrate or stress (Table 1). The initial sorting of the majority of these permeases is induced by their ubiquitylation catalyzed by Rsp5 Ub ligase with the requirement of ARTs [140].

Recent studies showed that some nutrient transporters belonging to the APC superfamily (AA and nucleobase transporters) are clustered in specialized PM domains called membrane compartments containing the arginine permease Can1 (MCCs) [142]. MCCs are PM furrows, ~50 nm deep and ~200–300 long, associated with subcortical structures called “eisosomes”, a reason why MCCs are also known as eisosome membrane compartments (EMCs) [42]. MCCs also contain the tetraspan TM proteins, Sur7 and Nce102 [143] and the core components of eisosomes are two self-assembling BAR-domain-containing proteins Pil1 and Lsp1 [144]. While Nce102 is essential for the formation of the furrow-like invaginations [143], Pil1 is responsible for its stabilization [144], [145], [146] (see Fig. 5A). Surprisingly, the lipidic composition of MCCs is not completely consensual. The majority of the studies has reported that these structures are enriched in ergosterol and sphingolipids based in the indirect evidence that MCC structure is affected by sphingolipids depletion, which was suggested to be sensed by specific proteins of MCCs (Nce102 and Slm1) [147], [148], [149] However, recent works, using a novel approach that enables the direct and *in situ* detection of the lipids surrounding a given PM protein, demonstrated that MCCs containing the AA transporters Lyp1 or Can1 have reduced levels of sphingolipids and that ergosterol is not so abundant as expected [150], [151]. Specifically, the authors of these works demonstrated that MCCs containing Lyp1 seem to be enriched in anionic lipids (such as phosphatidylserine) with saturated and unsaturated acyl chains and a small, but essential, amount of ergosterol [151]. Other recent study supports that MCCs are regions devoided of sphingolipids [152]. However, despite these findings, the lipidic composition of MCCs and of other PM domains is quite complex and is not completely conclusive yet. Therefore, further studies are required to completely elucidate this topic.

Depending on the nutrient status of the cell, transporters can move from MCCs to other regions/domains of the PM [153], [154]. MCCs may represent a novel endocytic regulatory mechanism, as they seem to protect transporters from internalization during nutrient limitation or starvation conditions, probably by preventing their access to the ubiquitylation machinery or even the formation of endocytic vesicles [42], [143], [147], [154]. Despite these findings, the molecular basis of PM transporter MCC partitioning is still elusive.

### 4.1 Endocytosis of amino acid transporters triggered by nitrogen availability or excess of substrate

In *S. cerevisiae*, different AA transporters undergo ubiquitin-dependent endocytosis in response to N availability or excess of substrate, including: Gap1, the General AA permease [155]; Can1, the high affinity arginine (Arg) permease [3], [15], [23]; Lyp1, the lysine (Lys) transporter; Mup1, the high affinity methionine (Met) permease [15]; Dip5, the aspartic acid (Asp) and glutamic acid (Glu) permease [156], [157] and Tat2, the tryptophan (Trp) and tyrosine (Tyr) permease [16].

**Fig. 4:**
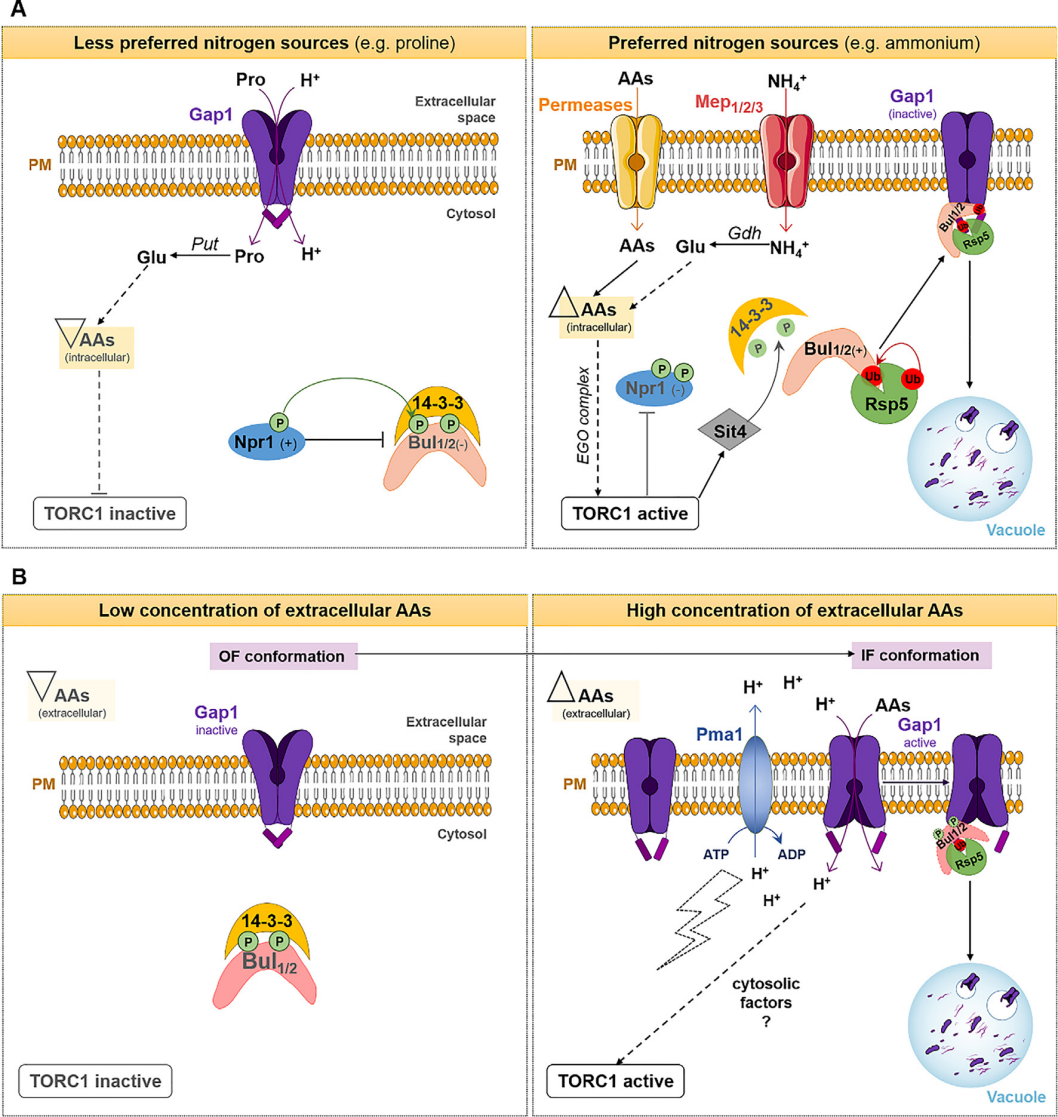
Schematic representation of Gap1 endocytic pathways. During growth on non-preferred nitrogen sources (such as proline), Gap1 is stable and localized at the plasma membrane (PM). However, if a preferred N source is added (e.g., NH_4_^+^), Gap1 is rapidly internalized, which can be induced via two distinct pathways: (A) Activity independent-Gap1 endocytosis (induced by intracellular AAs). In this pathway, NH_4_^+^ is imported via Mep permeases and then converted to glutamate (the major N donor) by glutamate dehydrogenases (Gdh) enzymes. Glutamate, in turn, promotes an increase in the concentration of intracellular AAs, which activates the TORC1 signaling via the EGO complex. Once activated, TORC1 inhibits Npr1 kinase, by promoting its hyper-phosphorylation, and activates Sit4 phosphatase. Sit4 dephosphorylates Bul1/2 protein adaptors and, consequently, causes their dissociation with 14–3-3 proteins. Once free of the inhibitory action of 14-3-3 proteins, Bul1/2 are ubiquitylated by Rsp5 ubiquitin ligase. Lastly, the complex Bul1/2-Rsp5 triggers ubiquitylation of Gap1, causing its internalization and further degradation in the vacuole [30], [171] (B) Activity dependent-Gap1 endocytosis (induced by extracellular AAs). In this pathway, internalization of Gap1 is dependent on substrate transport. AAs are imported through Gap1, which causes a transition of Gap1 from an OF to an IF conformation and exposes important residues that are further recognized by the ubiquitylation machinery (Bul1/2 and Rsp5) [45]. Recent studies suggest that the influx of protons (H^+^) coupled to AAs import represents a general signal for the activation of TORC1 complex [163]. AAs, amino acids; PM, plasma membrane; OF, outward-facing; IF, inward-facing; Ub, ubiquitylation; P, phosphorylation; NH4^+^, ammonium; H^+^, proton; ATP, adenosine-triphosphate; ADP, adenosine-diphosphate. Dashed lines represent predicted regulation; signals (+) and (−) represent activation and inhibition, respectively; upward- and downward-facing triangles represent increase and decrease of the substrate, respectively.

**Fig. 5:**
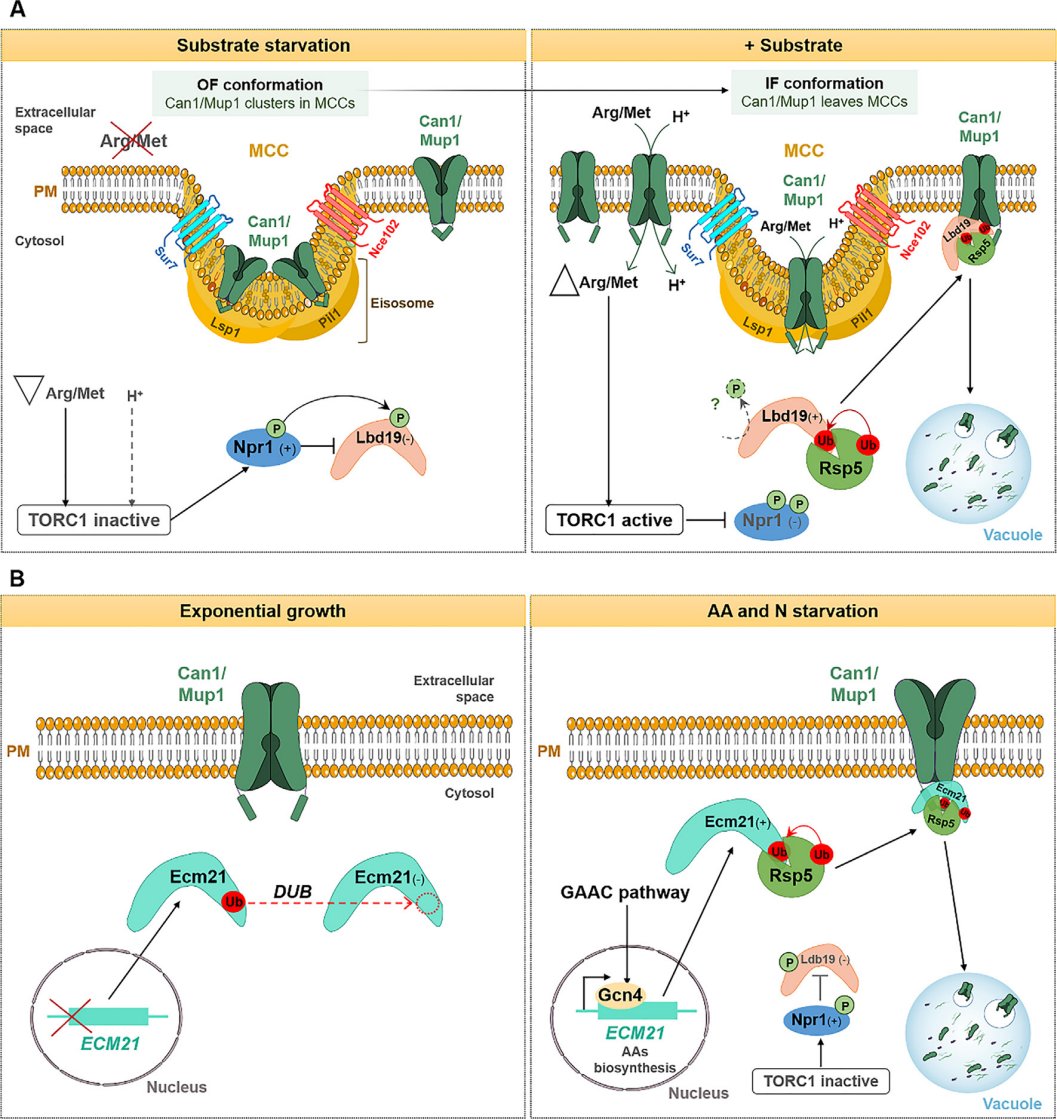
Schematic representation of Can1 and Mup1 endocytic pathways triggered by distinct signals. (A) Substrate-dependent downregulation of Can1 and Mup1. Under substrate starvation conditions (absence of arginine for Can1 and absence of methionine for Mup1), both Can1 and Mup1 preferentially localize at MCCs, presumably more populated in an OF conformation. Inside these domains, Can1 and Mup1 are protected from ubiquitylation machinery [42], [43], [154]. Low arginine/methionine concentrations maintain the inactive state of TORC1, stimulating Npr1 kinase, which, in turn, will phosphorylate Lbd19, leading to its inhibition [42], [43], [154]. If arginine/methionine is added, these AAs stimulate the TORC1/Npr1 pathway, which, in turn, leads to the formation of Ldb19/Art1-Rsp5 complexes. In parallel, the transport cycles of these AAs induce a transient shift of the transporters conformation, resulting in the diffusion of the transporter away from MCCs [42], [153], [154]. The IF conformation of these transporters exposes the N-terminal binding sites (degron) for Ldb19 adaptor, leading ultimately to Can1 and Mup1 ubiquitylation and subsequent degradation in the vacuole [42], [154]. (B) Starvation-induced downregulation of Can1 and Mup1. Under AAs or N starvation conditions, the GAAC pathway upregulates the *ECM21/ART2* gene by the action of Gcn4 transcriptional regulator, which causes an increase in Ecm21 protein levels and allows the subsequent formation of Ecm21-Rsp5 complexes. Ecm21-Rsp5 will then ubiquitylate Can1 and Mup1 transporters, inducing their endocytosis and degradation via the MVB pathway. Gcn4 also induces transcription of genes involved in *de novo* biosynthesis of AAs in order to keep AA homeostasis. Under rich growth conditions, *ECM21* transcription is strongly inhibited, which results in a decrease in the formation of Ecm21-Rsp5 complexes. Moreover, DUBs also appear to play a role in modulating Ecm21 activity, as already described for other transporters. PM, plasma membrane; OF, outward-facing; IF, inward-facing; MCC, membrane compartment containing the arginine permease Can1; Ub, ubiquitylation; P, phosphorylation; SL, sphingolipid; H^+^, proton; DUBs, deubiquitinating enzymes. Dashed lines represent predicted regulation; signals (+) and (−) represent activation and inhibition, respectively; upward- and downward-facing triangles represent increase and decrease of substrate, respectively.

Gap1, Can1 and Mup1, currently, have the best characterized endocytic mechanism (Fig. 4, Fig. 5).

Gap1 downregulation is controlled by N availability and excess of substrate. In the presence of a less preferred N source (e.g., proline or urea), Gap1 is localized at the PM. However, upon addition of a favored N source, that can be easily converted into the main AA precursors, like ammonium (NH_4_^+^), Gap1 is ubiquitylated on the K9 and K16 residues, located at the cytosolic N-terminus (Table 1). Then, it is subsequently internalized, directed to the MVB pathway and degraded into the vacuole [158]. This ubiquitylation depends on Rsp5 and Bul1 and Bul2 arrestins [159]. Gap1 downregulation occurs by excess of substrate and it is mediated by two different pathways. One is independent on Gap1 activity, as ubiquitylation also occurs in Gap1 activity defective mutants; and the other requires a functional Gap1 mediating substrate transport (Fig. 4).

In the first pathway, Gap1 is present at the PM when cells grow with proline as sole N source. Intracellular proline, imported via Gap1, is converted to glutamate by Put1 (proline oxidase) and Put2 (pyrroline carboxylate dehydrogenase) enzymes. The pool of glutamate resulting from proline conversion is less efficient, resulting in a lower concentration of intracellular AAs, being unable to activate TORC1 [160]. At these conditions, Bul1/2 proteins are phosphorylated (inactive) by the action of Npr1 kinase and associated with 14-3-3 proteins. If a preferred N source is provided, like NH_4_^+^, it is imported into the cells via NH_4_^+^ permeases (Mep1-2-3), converted into glutamate, by the Gdh1 and Gdh3 glutamate dehydrogenases, which is ultimately converted into AAs. The increase in intracellular AAs will then stimulates TORC1 signaling probably via EGO complex (reviewed in [160]). Once activated, TORC1 triggers hyper-phosphorylation and inhibition of the Npr1 kinase. Additionally, the Sit4 phosphatase induces dephosphorylation of the Bul1/2 proteins, promoting their release from 14 to 3-3 proteins and subsequent Gap1 ubiquitylation and internalization [30], [158], [161] (see Fig. 4A). Importantly, a cytosolic N-terminal region (20–35 AA residues) appears to be crucial for downregulation of Gap1 induced by intracellular AAs [162].

In the second pathway, Gap1 alters its conformation during substrate transport, transiently shifting from a substrate-free outward-facing (OF) conformation to an inward-facing (IF) conformation. This alteration induces the remodeling of its cytosolic regions, allowing its recognition by Bul1/2 adaptors and subsequent Rsp5-mediated ubiquitylation and degradation [45]. Notably, the stimulation of TORC1, which in this case is independent on intracellular AAs, is not exclusively linked to substrate transport, but also to the H^+^ influx coupled with AA import. Importantly, TORC1 activation in response to increased cytosolic H^+^ also requires the ATP-dependent proton pump Pma1 responsible for maintaining H^+^ gradient across the PM to avoid acidification of the cytoplasm and maintain the proton-motive-force. Replacement of Pma1 by an equivalent and catalytically active plant H^+^-ATPase in *S. cerevisiae* does not result in the stimulation of TORC1 in response to increased cytosolic levels of H^+^. These results suggest that Pma1 may modulate TORC1 via signaling, however, the molecular mechanisms connecting H^+^ to TORC1 activation remain elusive [163] (Fig. 4B). In contrast to other members of APC family, Gap1 is not localized in MCCs [43].

Additionally, O'Donnell and colleagues [20] showed that Gap1 traffic from endosomes to the TGN and/or the PM (see Fig. 1A). Under high N conditions, Gap1 transits from the TGN to the endosomes or vacuole without the involvement of the PM (see VPS pathway in Fig. 1). In contrast, when the levels of N decrease, the Gap1 recycling route from endosomes to the TGN/PM is activated (see endosome-to-Golgi retrograde and recycling pathways in Fig. 1A). This pathway requires Aly2/Art3 and Aly1/Art6 protein adaptors, which act in different directions. While Aly2 is responsible for Gap1 traffic from endosome-to-TGN (requiring AP-1, Lst4, and Npr1 proteins), Aly1 regulates Gap1 recycling from endosomes to the TGN and/or PM [20].

As mentioned above, some transporters of the APC superfamily were shown to be localized at MCC domains. It is the case of Can1 that preferentially localizes inside MCCs in the absence of its substrate. Can1 downregulation, triggered by excess of arginine, also relies on the TORC1-Npr1 cascade and is dependent on the transport activity of Can1 (Fig. 5A). Under poor N conditions, TORC1 is inactive and Npr1 causes the phosphorylation of Ldb19/Art1 arrestin, preventing its association with Can1 and subsequent ubiquitylation [3], [15], [45]. In the presence of arginine, TORC1 becomes active, inhibiting Npr1 kinase. Also, Can1 transiently swaps from an OF to an IF conformation, which triggers its movement out of MCCs, increasing its accessibility to ubiquitylation and consequent degradation [42], [153]. This conformational modification exposes Can1 binding sites for Ldb19 (70–81 AA residues), promoting Rsp5-mediated ubiquitylation, mainly on the K42 and K45 residues, and its subsequent degradation. The Can1 binding site for Ldb19 is rich in negative charged residues, also known as acidic patch [46] (Fig. 5A). Several studies suggest that activation of Ldb19 requires its dephosphorylation [3], [15], however, to date, no phosphatases were identified that promote Art1 dephosphorylation under arginine-repleted conditions. Moreover, in the absence of Ldb19, Bul1/2 are also able to ubiquitylate and elicit Can1 internalization. Yet, Bul1/2 bind to different regions of the N-terminus of Can1 (62–69 AA residues) and do not efficiently target the internalized Can1 to the vacuole (Table 1) [23].

The molecular mechanism that triggers transporter partitioning and exit from MCCs is not known yet. The Nce102 protein was proposed to be necessary for sequestration of Can1 into MCCs [143]. However, Gournas and colleagues demonstrated that removing *NCE102* was not sufficient to abolish Can1 MCC clustering [42]. Considering transporter exit from MCCs, other studies suggest that changes in Can1 conformation, during the transport cycle, can abolish the interactions of Can1 with specific lipids or proteins present in MCCs, which triggers its removal from these domains [42], [153]. A similar mechanism was also hypothesized for Mup1 [154]. Still, further research is needed to clarify these processes.

Endocytic downregulation of Lyp1, Mup1 and Tat2 transporters by excess of substrate (lysine, methionine, or tryptophan/tyrosine, respectively) also depends on the TORC1-Npr1 signaling, requiring the α-arrestin Ldb19 [15], [16], [46]. Similar to Can1, recognition of Mup1 by Ldb19 occurs in a specific region on the cytosolic N-terminus, the acidic patch (41–55 AA residues), and Mup1 is ubiquitylated on K27 and K28 residues. Nevertheless, additional features are needed for the efficient ubiquitin-dependent endocytosis of Mup1, because its N-terminus is required but not sufficient for its degradation [46]. Importantly, Lyp1, Mup1 and Tat2 are localized in MCC domains, a common feature shared by some members of the APC superfamily [43], [44], [154] and, as already described for Can1, crucial for their endocytic process, in response to nutrient status [42], [43], [44], [154], [164].

The aspartic acid/glutamic acid permease Dip5 is also endocytosed by substrate excess. O’Donnell and colleagues [157] demonstrated that dephosphorylation of Aly1 by the phosphatase calcineurin is required for Aly1-induced internalization of Dip5 permease and subsequent targeting to the vacuole [157]. It was also reported that Aly2 arrestin, functions as an adaptor of Rsp5 to mediate Dip5 ubiquitylation and downregulation. In this case, phosphorylation of the N-terminal tail of Dip5 (10–22) also contributes to its internalization (Table 1) [156].

Tight regulation of AA uptake via endocytosis in *S. cerevisiae* is likely related to AA biosynthesis pathways. In this microorganism, all N containing compounds are synthesized using either glutamate or glutamine as N donor [165], both of which can be synthesized using ammonia directly to supply the amino group [166], [167], [168]. Therefore, when *S. cerevisiae* is cultivated in any other N source, this has first to be converted to ammonia and glutamate, a process that can be limiting for cellular growth [169]. Therefore, tuning N transport, in a way that preferred N sources are preferentially selected, allows *S. cerevisiae* to achieve the highest growth rates in the presence of multiple N sources [170].

The endocytic regulation of AA transporters in *Aspergilli*, in particular in *A. nidulans* [19], [172] has also been reported. This organism contains 19 putative genes enconding transporters of the YAT family [173]. However, only 2 of these transporters were functional caracterized, namely, the AgtA high-affinity dicarboxylic AA transporter, responsible for the uptake of aspartate and glutamate, and the PrnB proline transporter [172], [174], [175], [176].

Both AgtA and PrnB are subjected to NH_4_^+^-induced downregulation [172], [173]. Specifically, AgtA and PrnB are localized at the PM, in cells grown in glutamate or proline containing medium, as sole N source, respectivelly. However, upon NH_4_^+^ addition, these transporters are internalized and targeted for vacuolar degradation [172], [173]. In these conditions, the internalization of PrnB requires ArtA, in contrast to what is described for AgtA [19].

In *A. oryzae*, the AoCan1 transporter (homologue of *S. cerevisiae* Can1) is localized at the PM, mainly in the basal region, when cells grow in arginine- starvation conditions. However this transporter is internalized and targeted to the vacuole for degradation, after a shift to a medium containing excess of arginine (5 mM) [177]. Contrarily, to *S. cerevisiae*, the ARTs and signalling complex(es) involved in arginine-induced downregulation of AoCan1 is/are still under investigation.

### 4.2 Endocytosis of amino acid transporters triggered by substrate depletion or acute starvation

Can1, Mup1, Lyp1 and Tat2 fAATs are also degraded in response to AAs or NH4^+^ starvation by a molecular mechanism distinct from the one involved in endocytic degradation triggered by excess of substrate [21], [49]. Substrate-induced downregulation of these 4 fAATs is faster, exclusive for its own substrate and relies on TORC1 signaling and Ldb19 adaptor [3], [15], [21], [42], [46], [154]. In contrast, internalization of fAATs elicited by AA and NH4^+^ starvation is a slower process (requiring 3–6 h); it depends on the general amino acid control (GAAC) pathway and requires specifically the Ecm21/Art2 arrestin. Additionaly, while Ldb19 recognizes an acidic patch localized at the N-terminus of the transporters, Ecm21 interacts with a C-terminal acidic degron [21] (Table 1; Fig. 5). The GAAC pathway induces the transcription of *ECM21* and of genes involved in *de novo* AAs synthesis, preparing the cells for non-selective nutrient acquisition, if nutrients become accessible again [21], [49] (Fig. 5B).

Overall, all the studies related to nutrient transporter (HXTs, AATs) endocytosis induced by starvation conditions seem to rely on the interplay of several nutrient signaling pathways (Snf1/AMPK; Ras/cAMP-PKA; TORC1; GAAC), culminating in the modulation of *ARTs* gene expression (Figs. 2A; 5B).

### 4.3 Endocytosis of amino acid transporters triggered by cell stress

Cycloheximide, rapamycin or PM structure disrupters, also induce the endocytosis of several AA transporters (Table 1). Lyp1 downregulation by cycloheximide requires Ecm21, in contrast to the lysine-induced internalization of Lyp1, which is dependent on Ldb19 adaptor [15]. Also, cycloheximide-induced endocytosis of Can1 requires the recruitment of Ldb19, which recognizes specific domains of the N-terminus of this permease, inducing its ubiquitylation, internalization and degradation [15]. Cycloheximide-triggered endocytosis of Tat2 depends on Ecm21, and to a less extent on Crs2 [16]. Importantly, rapamycin, heat shock, oxidative and alcoholic stresses also trigger ubiquitylation and downregulation of Gap1, in a rRsp5 dependent manner , involving Bul1, Bul2, Aly1 and Aly2 arrestins. These adaptors act mainly via C-terminus of Gap1 and remain phosphorylated and possible connected to 14-3-3 proteins. In contrast to Bul1/2 proteins, the Aly1/Aly2 arrestins induce ubiquitylation exclusively on the K-16 residue [178]. Can1 and Lyp1 permeases also undergo internalization in response to an increase in temperature or pH, a decrease in osmolarity, or the presence of amphiphilic compounds. Yet, the arrestins behind these processes have not been identified [178]. Notably, these stress conditions, which lead to an increase in PM fluidity, have been associated with MCCs disassembly and, consequently, result in increased ubiquitylation and degradation of some APC permeases (reviewed in [43], [44]). Membrane fluidity is partly governed by the crowding effect of macromolecules, including transport proteins, and therefore likely influenced by endocytosis of membrane transporters [179]. Since PM fluidity is a major determinant in yeast stress tolerance [180], endocytosis of AA transporters, which are not directly linked to energy provision, could have evolved as a strategy to increase phenotypic diversity in a stressed population to increase the chance of surviving individuals.

## 5 Endocytosis of nucleobase transporters

In Fungi, pyrimidines and purines nucleobases transporters are divided in three distinct families, namely the Nucleobase/Cation Symporter 1 (NCS1) family [181], [182], [183], the Nucleobase Ascorbate Transporter (NAT) family (also named nucleobase cation symporter family 2 (NCS2)) and the AzgA-like family [184].

Transporters belonging to these families also undergo endocytic downregulation in response to different environmental signals. Examples include the pyrimidine Fur-like transporters from *S. cerevisiae* and *A. nidulans* and the purine transporters UapA, UapC and AzgA from different *Aspergillus* species.

### 5.1 Endocytosis of Fur-like transporters

**Fig. 6:**
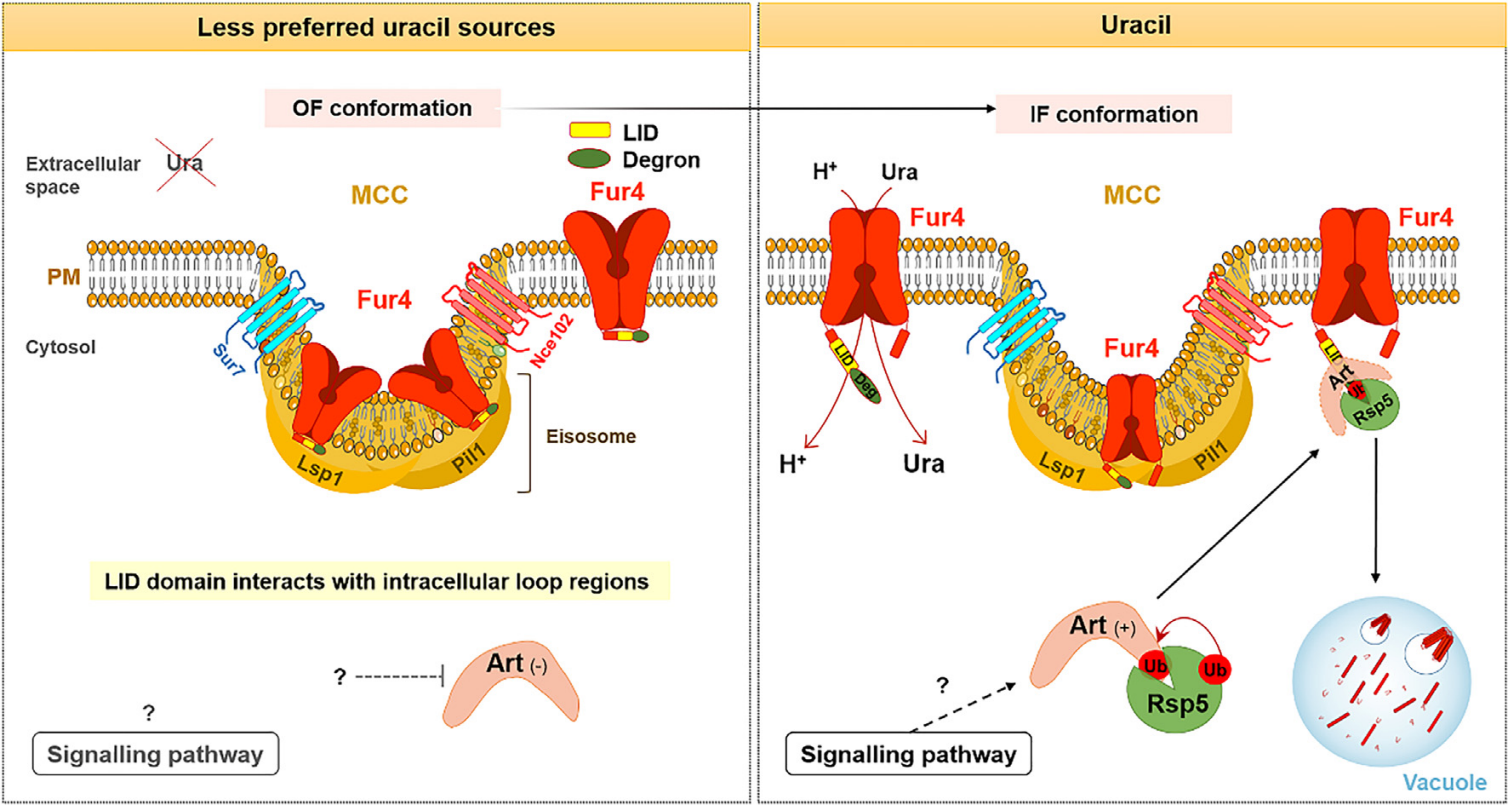
Schematic representation of Fur4 regulated endocytosis. Under uracil starvation conditions, Fur4 is localized at the PM, preferentially inside the MCCs [43] and the LID sequence is in close contact with the intracellular loops of Fur4. Addition of uracil elicits conformational changes sensed by the LID, triggering Fur4 exit from MCCs and exposing the degron sequence to Art-Rsp5 complexes. This ultimately leads to Fur4 ubiquitylation, internalization and degradation via the MVB pathway [41], [43], [189], [190]. PM, plasma membrane; Ura, uracil; OF, outward-facing; IF, inward-facing; MCC, membrane compartment containing the arginine permease Can1; Ub, ubiquitylation; P, phosphorylation; ARTs, arrestin-related trafficking adaptors; LID, loop interaction domain; H^+^, proton. Dashed lines represent predicted regulation and signals (+) and (−) represent activation and inhibition, respectively.

The *S. cerevisiae* uracil:cation symporter Fur4, belonging to the NCS1 family, is one of the most extensively-studied transporters at the level of regulation of cellular expression [185]. The structure of Fur4 and other Fur-like transporters, is based on the crystal structure of the Mhp1 bacterial homologue. These transporters are composed of 12 TMS, the first 10 are involved in transport catalysis, while the role of the last two remains elusive. The transport associated with the 10 TMSs form two inverted repeats arranged in a two-fold pseudosymmetrical axis and oppositely orientated with respect to the PM. The N- and C-termini of all Fur transporters are oriented towards the cytoplasm [41], [186], [187], [188]. Importantly, the Fur4 N-terminal region contains a degron motif, ubiquitylation acceptor sites K31 and K41, a PEST-like sequence, and a Loop Interaction Domain (LID). The LID sequence senses conformational changes in the permease, and the degron is involved in the accessibility to the ubiquitin acceptor lysines, being required for ubiquitylation and endocytosis of Fur4 (see Fig. 6) [41], [189], [190].

Moreover, Fur4 was also found to be enriched in MCCs [147], [191], [192] and, similarly to other mentioned members of APC superfamily, association of Fur4 with these domains preferentially stabilizes this transporter at the PM, in cells growing under low uracil conditions [43].

Internalization of Fur4 is induced by both intracellular and extracellular uracil [41], [193], as well as by stress conditions that disrupt PM lipid structure or H^+^ flux (e.g., heat shock, peroxide, or alkali stress) [27], [43] (Table 1).

Particularly, under uracil starvation conditions, Fur4 is stable at the PM and preferentially localizes at MCCs [43]. Under these conditions, the N-terminal LID sequence is in close contact with intracellular loops of Fur4. However, upon addition of uracil, each transport cycle induces conformational changes (transiently shifts from an OF to an IF conformation), which are sensed by the LID, exposing its degron to ARTs and causing Fur4 internalization and degradation. The interaction with the ubiquitylation machinery occurs outside MCCs, allowing the access of Art-Rsp5 complexes [41], [43], [189], [190]. The signaling complexes/pathways behind Fur4 endocytosis remain to be elucidated (Fig. 6). This proposed LID-degron mechanism, operating by the N-terminus portion, is also supported by studies in Gap1 [162] and in bacterial LeuT transporter [194]. Despite high uracil selectivity, Fur4 exhibits a degree of redundancy in terms of α-arrestin recognition [16].

Fur4 endocytosis triggered by AA/NH4^+^ starvation was also reported, but this process does not depend on Ecm21 arrestin, in contrast to what was described for AATs [21]. Further details on the molecular mechanisms need to be clarified.

In *A. nidulans*, Fur-like transporters, homologues of *S. cerevisiae* Fur4 and other members of the NCS1 family, have been extensively studied in relation to function, specificity, cellular expression and evolution [13], [38], [39], [187], [188], [195].

In particular, FurA (high-affinity allantoin transporter), FurD (high-affinity uracil transporter) and FurE (low affinity transporter of uracil, allantoin, uric acid, and related analogs) show endocytic turnover in response to NH_4_^+^ or excess of their substrates [13], [187], [188], a process requiring their cytosolic terminal domains [38], [39]. Indeed, dynamic interaction of cytosolic FurE termini with each other seems to play a role in its endocytosis, in a mechanism dependent on transport activity of FurE. The proposed model suggests that, in the absence of substrates, FurE C- and N- cytoplasmic termini are in close contact. However, upon substrate addition, FurE transiently switches from an OF to an IF conformation, exposing residues/motifs that are further recognized by the endocytic machinery [38]. Genetic and mutational analysis demonstrated that FurE, similarly to Fur4, also contains a LID motif crucial for specificity but, unlike Fur4, not essential for endocytosis. Instead, FurE internalization requires, in addition to K521 and K522 as ubiquitylation sites, a short acidic C-terminal sequence (501–503 AA residues; a possible ART-binding site) and elements present in the distal part of the N-terminus (1–21 AA residues) [5], [39].

### 5.2 Endocytosis of the high-affinity purine transporters

Purine transporters that are known to be regulated by endocytosis include the high-affinity purines H^+^ symporters: UapA from *A. nidulans* [182], [196], UapC from *A. nidulans* [197] and *A. oryzae* [198], [199], all belonging to the NAT/NCS2 family, and AzgA from *A. nidulans* [19], member of the AzgA-like family.

In contrast to filamentous fungi, *S. cerevisiae* does not possess protein members of the NAT/NCS2 [181] or the AzgA families [181], [184].

UapA is a high-affinity, high-capacity uric acid/xanthine H^+^ symporter, containing 14 TM segments organized into two domains, a gate domain (TMs 5-7, 12-14) and a core domain (TMs 1-4, 8-11) [200].

The endocytosis of UapA transporter has been extensively characterized in *A. nidulans* [182], [196]. When UapA is at the cell surface, it is ubiquitylated by HulA^Rsp5^ E3 ubiquitin ligase in response to excess of substrate (xanthine or uric acid) or presence of preferred N sources (NH_4_^+^ or glutamine). This requires a highly selective ubiquitin acceptor residue (K572) within the C-terminal region of UapA [18]. Ubiquitylated UapA is, then, endocytosed and directed to the MVB for vacuolar degradation. The substrate-elicited endocytosis of UapA is dependent on UapA transport activity, unlike NH_4_^+^-elicited endocytosis [18]. A di-acidic motif (545-EVE-547) in UapA C-terminal region (Table 1) is also critical for ArtA-mediated ubiquitylation and endocytosis of UapA induced by both signals: addition of substrate or ammonium [19]. Also, it was shown that N-terminal motifs of UapA, as well as the interaction of UapA with lipids of the PM, are crucial for its oligomerization, traffic and function [201], [202], [203]. As already mentioned, UapA endocytosis, but also that of several other studied nutrient transporters in *A. nidulans*, was proved to be clathrin-dependent but independent of AP2, which is considered to be the standard partner of clathrin [55] (see Fig. 1B).

UapC is a high-affinity, moderate-capacity, uric acid-xanthine transporter, but also imports hypoxanthine, adenine, and guanine with lower affinity [204]. In both *A. nidulans* and *A. oryzae* species, UapC is localized at the cell surface in cells growing in urea as sole N source, but if NH_4_^+^ is added to the medium, the transporter is removed from the PM and targeted to the vacuole, after a few minutes [197], [198], [199].

AzgA is an adenine-hypoxanthine–guanine H^+^ symporter that contains 14 putative TMSs and cytoplasmic N- and C-termini [184]. AzgA localization at PM is not affected by addition of NH_4_^+^, however, this transporter undergoes substrate-induced downregulation with the requirement of the ArtA adaptor [19]. Similar to UapA transporter, the cytosolic termini of AzgA also contain di-acidic motifs. Still, their role in AzgA endocytosis has not been addressed yet [19].

The reported studies advanced the knowledge on the endocytosis of purine transporters in *Aspergillus* species, still more studies are required to understand the signaling pathways and proteins involved in this process.

## 6 Endocytosis of metal micronutrient transporters

Metal ions such as copper (Cu), iron (Fe), zinc (Zn) and manganese (Mn) are essential micronutrients for all living organisms. They function as co-factors for different enzymes that participate in crucial biological processes with impact on growth, metabolism and physiology of yeast. Nevertheless, when present at high levels, these micronutrients are toxic (reviewed in [205], [206], [207]).

In *S. cerevisiae,* several PM metal transporters are reported to be regulated by endocytosis, including the high-affinity Cu transporter Ctr1, the high-affinity reductive Fe transport system Fet3-Ftr1, the high-affinity Zn transporter Zrt1, and the broad-specificity metal ion transporter Smf1 [58], [60], [106], [208], [209], [210], [211], [212], [213], [214] (Table 1).

To maintain a constant proton-motive force (pmf) with changes in extracellular pH, in many organisms a charge difference (*Δψ*) over the PM is established [215]. At pH values above ~ 3, representing the physiological conditions of *S. cerevisiae*, this *Δψ* results in an intracellular environment that is negatively charged compared to the outside [216], [217], [218], providing a driving force for protons and other cations such as metal micronutrients. Therefore, the rapid internalization of metal and other cation transporters is an efficient cellular mechanism involved in metal-ion homeostasis, and it is likely to play an important role to prevent toxic intracellular accumulation of these compounds in *S. cerevisiae*. Regarding *Aspergilli*, to the best of our knowledge, the endocytic regulation of metal ion transporters has not been reported in these organisms.

### 6.1 Endocytosis of copper transporters

Cu import is mediated by the high-affinity Ctr1 transporter. Studies based on human Ctr1 [219], [220] suggest that *S. cerevisiae* Ctr1 is composed by three monomers which are organized in a channel-like architecture. Each monomer is composed by 3 TMSs, an extracellular N-terminus (rich in multiple methionine motifs), and a cytosolic C-terminal domain [221], [222], [223].

In the presence of Cu-limiting concentrations (medium lacking Cu or containing Cu-chelating compounds), Ctr1 is localized at the PM, however, upon extracellular Cu addition (50 µM CuSO_4_), Ctr1 is rapidly endocytosed and delivered to the vacuole for degradation [208]. Ctr1 internalization appears to be preceded by its Rsp5 mediated ubiquitylation at K340 and K345 residues (present at the C-terminus), which seems to be mediated by the ARTs Bul1/2 [208]. Endocytosis of Ctr1 does not appear to require an active transporter, since a mutant version lacking detectable Cu transport activity was still ubiquitylated, internalized and degraded [208]. A subsequent work by Wu and collaborators [224] challenged the view that Ctr1 is endocytosed by excess copper. This report, which did not include fluorescence microscopy assays, was based on genetic analysis using mutants in the endocytic pathway and a *rsp5/npi* mutant. The authors propose, instead, that, in the presence of excess copper, Ctr1 is rapidly inhibited upon copper binding, by a structural remodeling mechanism, dependent on the C-terminal cytosolic tail of Ctr1 [224]. However, Liu and co-workers [208] used GFP under the control of a *CTR1* native promoter [208], whereas Wu et al. studied Ctr1-GFP expression under a *TEF2* promoter [224], which most likely resulted in the overexpression of the transporter. These and other differences in the experimental procedures could account for the conflicting reports, which need further clarification.

### 6.2 Endocytosis of iron transporters

In *S. cerevisiae*, the high-affinity iron (Fe) uptake complex, Fet3-Ftr1, consists of the multicopper oxireductase Fet3 (which oxidizes ferrous Fe^2+^ to ferric iron Fe^3+^) and of the ferric iron permease Ftr1, responsible for the subsequent uptake of Fe^3+^. The protein structure of Ftr1 is predicted to contain 7 TMSs, while the Fet3 only contains 1 TMS. Both proteins contain an extracellular N-terminus and a cytosolic C-terminus, with both C-termini in close proximity [225], [226]. Studies from Kwok and colleagues support the idea that Fet3p-Ftr1p function in a channeling mechanism [227].

This heterodimeric complex is constitutively internalized when yeast cells grow under intermediate concentrations of Fe (10–100 µM); however, a dynamic population of Fet3-Ftr1 is kept at the PM via endocytic recycling [210] (see Fig. 1A, Endosome-to-PM recycling pathway). An endocytic recycling motif (319–328 AA residues) present at the C-terminus of Ftr1 was identified and seems to be specifically recognized by a sorting nexin (Grd19p/Snx3p) that functions as an adaptor to link protein cargo to the cellular recycling machinery (e.g., retromer complex) [210]. In contrast, addition of high Fe concentrations (1.0 mM) to Fe-starved cells (growing in a medium containing the Fe chelator bathophenanthroline disulfonate (BPS)) triggers the internalization and vacuolar degradation of the entire complex, in a mechanism dependent on Fet3-Ftr1 ubiquitylation and on an active Fe transport system, [209]. It seems that the fate of this protein complex (i.e., its recycling or degradation) is dependent on Fe levels. However, further studies are needed to clarify these processes.

### 6.3 Endocytosis of zinc transporters

In Zn shock conditions, Zn homeostasis is reported to depend mostly on the increased expression of the Zrc1 vacuolar Zn transporter but also on the regulated endocytosis of the PM high-affinity Zn transporter Zrt1. This transporter contains 8 predicted TMSs, with both N- and C-termini localized in the extracellular space and a very large intracellular loop connecting the TMS3 with TMS4 [228], [229]. When Zn-starved cells (growing with Zn chelator EDTA) are treated with high concentrations of extracellular Zn (1 µM-2 mM), Zrt1 is ubiquitylated at K195 (localized in the large cytoplasmic loop between TMS3 and TMS4), internalized from the PM and targeted for vacuolar degradation [211], [212], [213]. A region between TM3 and TM4 (205-211 AA residues) seems to be crucial for Zrt1 ubiquitylation and inactivation. However, the fusion of this region to other PM transporters, such as Pma1 and Irt1, does not result in their ubiquitylation and degradation, implying that this Zrt1 domain must adopt a certain conformation to be functional [213]. Endocytic inactivation of Zrt1 seems to, not only increase tolerance to Zn, but also to the non-essential toxic metal cadmium, a substrate for Zrt1, which also triggers the transporter degradation. This process protects cells from cadmium accumulation and toxicity, preventing its uptake by Zrt1 [211], [213].

### 6.4 Endocytosis of a broad-specificity metal ion transporter

Smf1 is a divalent metal ion transporter responsible for the uptake of manganese, but also Fe, Cu and other metals like cadmium, cobalt and nickel. This high-affinity transporter is present at the PM in manganese starvation conditions [106]. Several signals have been demonstrated to trigger Smf1 endocytosis and degradation, which include physiological concentrations of Mn (described as non-toxic metals concentrations) [214], [230], toxic levels of Mn [60] and cadmium [106]. When cells grown in Mn starvation conditions are treated with physiologic Mn levels (5–10 µM), a bulk of Smf1, but not all of the protein, is degraded in the vacuole in a mechanism dependent on the E3 Ub ligase Rsp5, the Transferrin-REceptor like proteins Tre1/2 and the membrane protein Bsd2. The ongoing degradation of Smf1p keeps the transporter at a low level, preventing the transport of potentially toxic metals while sufficient for essential manganese uptake [231]. This model of “basal MVB sorting” proposes that, within the cell, much of the newly synthetized Smf1 is recognized by a complex formed by Tre1/2 and Bsd2 adaptors, which have PPxY motifs, triggering Rsp5-mediated ubiquitylation of Smf1. Ubiquitylated Smf1 enters the MVB pathway and is delivered to the vacuole where it is degraded [60], [214], [232]. Under Mn starvation conditions, the Tre1/2-Bsd2 complex fails to recognize Smf1 and this transporter is directed to the cell surface [214].

Toxic levels of Mn (>5 mM) also lead to Smf1 degradation [60], including both cell surface Smf1 endocytosis and intracellular Smf1 degradation via the VPS pathway (from the Golgi to the MVB, see in Fig. 1) but independent of Bsd2/Tre proteins. While Smf1 downregulation triggered by physiological Mn is dependent on metal transport activity, Smf1 downregulation during chronic Mn toxicity is not dependent on a functional or active transporter [60], [214].

Cellular exposure to toxic cadmium ions (0.1 mM cadmium chloride) also induces Smf1 downregulation. This process is dependent on Smf1 ubiquitylation, which is mediated by the Rsp5 ubiquitin ligase and Crs2 or Ecm21 arrestin proteins. Phosphorylation of Smf1 at the N-terminus is required for binding of Ecm21, although this post-translational modification does not seem to be triggered by Cd stress. After binding, Ecm21 recruits Rsp5 Ub ligase, which leads to Smf1 ubiquitylation, at K33 and K34 residues, and its subsequent targeting for vacuolar degradation [106].

Besides the substrate-induced internalization of metal transporters, AA and N starvation conditions have also been shown to trigger endocytosis of Ctr1 and Ftr1 metal transporters [21], [49]. This process also requires Rsp5-mediated ubiquitylation but it is independent of TORC1 pathway [49]. Further experimental evidences are needed to confirm whether or not starvation-induced downregulation of Ftr1 and Ctr1 relies on the GAAC pathway, as observed for some fAATs (Can1, Mup1, Lyp1 and Tat2) [21].

## 7 Biotechnological relevance of nutrient transporter endocytosis

The concept of the cell factory is central to microbial biotechnology. Cell factories have been designed for the production of both bulk and high value products, food processing, and the production of pharmaceuticals. In the past decades, different strategies have been progressively developed to improve the design of these cell factories (reviewed in [233]). The capacity to experimentally modify the steady-state/activity of nutrient transporters at the PM could be an important complementary strategy [234], as these are critical for the concentration of substrates inside the cell, for the secretion of products by the engineered strains and have the potential to impact the titer, rate and yield (TRY) of industrial processes. To achieve high titers (T) without reaching toxic intracellular concentrations, the final production phase in processes for molecules such as organic acids benefits from the use of energy-driven transport systems [216]. Secondly, limited space in plasma membranes [98], [99] and competition for this space between endogenous and overexpressed transporters [100] has the potential to limit the maximal activity of the engineered transport systems. Therefore, to achieve the highest possible conversion rates (R) of substrate to product, it is important that transporters are only expressed and present at the PM when required for product synthesis or for an (essential) metabolic function. Energetic coupling of uptake, conversion and export of substrates and products often impacts the overall product yield [235]. Therefore, replacement of energy-dependent transport systems by facilitated diffusion has the potential to substantially increase product yields (Y) in processes where substrate is abundant and/or the product non-toxic [236].

As exemplified in this review, endocytosis of transport proteins plays an important role in survival under the dynamic growing conditions found in nature. Similarly, in the inherently dynamic conditions found in industrial fed-batch processes, understanding and targeted engineering of endocytosis is an essential step towards the establishment of robust industrial microorganisms. For example, improved stabilization of specific *S. cerevisiae* glucose transporters could prevent ATP expenditure by limiting their turnover due to dynamic conditions found in large-scale bioprocesses [237]. Other studies suggested that stabilization of glucose transporters at the PM, after glucose depletion, could be an optimization strategy to facilitate the use of other C sources [234], [238].

Furthermore, identification of specific α-arrestins, that mediate PM nutrient transporters turnover or recycling, as well as the specific motifs of the transporter where α-arrestins bind, may allow the design of more stable strains by either maximizing the uptake of specific nutrients or the extrusion of the unwanted metabolites or products of interest. For instance, mutation of *CreD*, which codes for an arrestin-like protein, inhibited the glucose-induced endocytosis of MalP, a major maltose permease of *A. oryzae*, ultimately resulting in increased production of amylolytic enzymes [239].

*Aspergillus* species are of great biotechnological importance for the production of high-value industrial and medical products (such as enzymes, organic acids and secondary metabolites) (reviewed in [240]). Therefore, expanding our knowledge on the *Aspergillus* fungi, namely in the field of nutrient transporters, post-translational regulation and in the identification of the proteins involved in this process, may widen the molecular toolbox available to the metabolic engineer.

Few reports combine robust physiological data with cell biology analysis. An improved and integrated understanding of these processes is relevant from a fundamental point of view (given the role of transporters as key sources of C, N and energy for microorganisms) but also from an applied perspective, considering their potential for the biotechnological field.

There is surely much untapped potential for the continued engineering of post-translational regulation of nutrient transporters and a strong motivation for the characterization of the proteins involved.

## 8 Conclusions and future perspectives

Endocytosis has a physiological role in the homeostasis of nutrients, and the mechanisms underpinning the reciprocal regulation of endocytosis and metabolism are now being unveiled.

The multitude of nutrient transporters known to be regulated by endocytosis highlights the critical importance of this regulation for the adaptation of cells to their environment and, consequently for their existence.

**Fig. 7:**
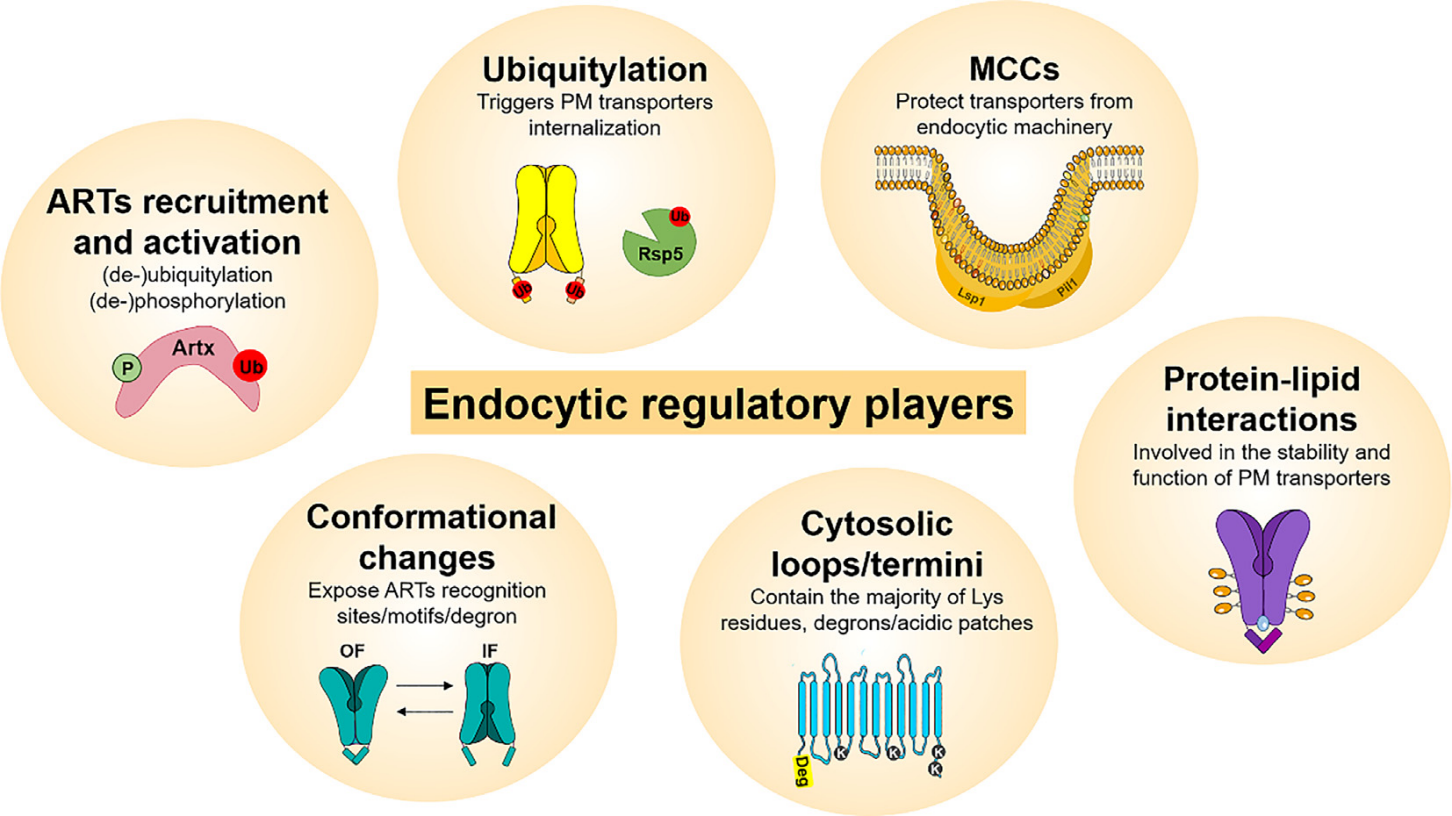
Summary of the main players, modifications and mechanisms involved in the regulation of nutrient transporters endocytosis.

Endocytosis is regulated on several levels, including posttranslational modifications, recruitment of adaptor proteins, substrate transport, conformational changes, dynamic interaction between cytosolic termini and/or their loops, as well as the proper interaction and organization of the transporters within specific domains (e.g., MCCs) of the PM lipid bilayer (Fig. 7). The many regulatory levels also reinforce the notion that this process must be fine-tuned for cell survival.

The dysregulation of genes involved in the endocytic pathway is associated with a wide array of growth defects, connected to the loss of ability to quickly respond and adapt to nutrient fluctuations or stresses.

Further investigations are necessary to clarify the roles of each regulatory circuit and how they communicate with each other and possibly with yet unidentified regulators. The continued study of the function and regulation of PM eukaryotic nutrient transporters is invaluable for their broad application as targets in biotechnological and medical fields, including their use as potential drug delivery systems in future therapies.

## CRediT authorship contribution statement

**Cláudia Barata-Antunes:** Writing - original draft, Writing - review & editing, Conceptualization, Visualization. **Rosana Alves:** Writing - original draft, Writing - review & editing, Conceptualization. **Gabriel Talaia:** Writing - original draft. **Margarida Casal:** Writing - review & editing. **Hernâni Gerós:** Writing - review & editing. **Robert Mans:** Writing - original draft, Writing - review & editing, Conceptualization. **Sandra Paiva:** Writing - original draft, Writing - review & editing, Conceptualization, Supervision.

## Declaration of Competing Interest

The authors declare that they have no known competing financial interests or personal relationships that could have appeared to influence the work reported in this paper.

## Footnotes

1 NEED4 - neural precursor cell expressed developmentally, down-regulated protein 4

2 HECT - homologous to the E6-AP carboxyl terminus

3 2% (w/v) raffinose concentration is equivalent to 0.2% (w/v) glucose [247].
